# Common Variants in *LRP2* and *COMT* Genes Affect the Susceptibility of Gout in a Chinese Population

**DOI:** 10.1371/journal.pone.0131302

**Published:** 2015-07-06

**Authors:** Zheng Dong, Dongbao Zhao, Chengde Yang, Jingru Zhou, Qiaoxia Qian, Yanyun Ma, Hongjun He, Hengdong Ji, Yajun Yang, Xiaofeng Wang, Xia Xu, Yafei Pang, Hejian Zou, Li Jin, Jiucun Wang

**Affiliations:** 1 Ministry of Education Key Laboratory of Contemporary Anthropology, Collaborative Innovation Center for Genetics and Development, School of Life Sciences, Fudan University, Shanghai, China; 2 Division of Rheumatology and Immunology, Changhai Hospital, Shanghai, China; 3 Division of Rheumatology, Renji Hospital, Shanghai Jiaotong University School of Medicine, Shanghai, China; 4 Division of Rheumatology, Taixing People’s Hospital, Jiangsu Province, China; 5 Division of Rheumatology, Taizhou People’s Hospital, Jiangsu Province, China; 6 Fudan-Taizhou Institute of Health Sciences, Taizhou, Jiangsu Province, China; 7 Division of Rheumatology, Huashan Hospital, Fudan University, Shanghai, China; 8 Institute of Rheumatology, Immunology and Allergy, Fudan University, Shanghai, China; IPK, GERMANY

## Abstract

Gout is a common inflammation disease resulting from an increase in serum uric acid. Nearly 70% of uric acid is excreted via the kidneys. To date, evidence for an association between genetic loci and gout is absent, equivocal or not replicated. Our study aims to test variants in two genes abundantly expressed in the kidney, *LRP2* and *COMT*, for their association with uric acid and gout. In total, 1318 Chinese individuals were genotyped for rs2544390 in *LRP2* and rs4680 in *COMT*. These *LRP2* and *COMT* gene polymorphisms showed no significant effect on uric acid (*P* = 0.204 and 0.188, separately); however, rs2544390 in *LRP2* did influence uric acid levels in individuals with BMI ≥ 25 (*P* = 0.009). In addition, the allele frequency distributions of the two loci showed a significant difference between gout patients and healthy controls. A missense variation in rs4680 (G > A) decreased the risk of gout (OR = 0.77, *P* = 0.015), whereas the T allele of rs2544390 was associated with gout pathogenesis risk (OR = 1.26, *P* = 0.020). The present study provides the first evidence for an association between *COMT* and gout. Rs2544390 in *LRP2* only influenced uric acid levels in individuals with BMI ≥ 25, which might explain the discrepant results among previous studies. In addition, we are the first to identify the association between *LRP2* and gout in a Chinese population and to confirm this association in Asians.

## Introduction

Gout is a common inflammation disease resulting from an increase in serum uric acid. Urate is synthesized mainly in the liver, and approximately two-thirds of daily urate excretion occurs via the kidneys [1, 2]. A decrease in the renal disposal rate of urate leads to nearly 90% of gout cases [3]. Over the past several years, evidence linking uric acid to some factors, such as body mass index (BMI), insulin resistance, metabolic syndrome, renal disease and hypertension, has been reported [4]. A genome-wide association study (GWAS) identified common genetic variations in 28 loci associated with uric acid [5]; however, this only explained 7% of the variance in serum uric acid concentrations, and only a portion of the variations were reported to be associated with gout [5, 6]. Therefore, it is necessary to identify novel candidate loci that may be associated with serum urate and gout. Catechol-O-methyltransferase (COMT), which is abundantly expressed in the liver and kidney, catalyzes the transfer of a methyl group from S-adenosylmethionine to catecholamines and plays an important role in regulating renal dopamine activity [7, 8]. Dopamine has an effect on the regulation of several renal functions, such as glomerular filtration and sodium excretion [7], and dopamine-induced glomerular filtration has been reported to be correlated with uric acid concentrations and urate excretion [9]. Thus, *COMT* may be a candidate gene in the pathogenesis of gout.

In addition, a recent GWAS in a Japanese population suggested the association between serum urate and rs2544390, a single-nucleotide polymorphism (SNP) in the intron of the low-density lipoprotein-related protein 2 (*LRP2*) gene [10], which is abundantly expressed in the kidney. However, another GWAS showed no significant association between rs2544390 and uric acid levels [11]. Therefore, it is urgent to test these loci for associations with uric acid and to determine the potential reasons for the equivocal results. With regard to gout, the population-specific effects at *LRP2* have been proven, i.e., rs2544390 polymorphism is associated with a risk for gout in a combined Māori and Pacific Island cohort. Conversely, the opposite was found in European subjects [6], suggesting the necessity for transancestral studies, especially in populations with no related studies, such as Chinese.

In the present study, we aimed to test variants in the *LRP2* and *COMT* genes, both of which are abundantly expressed in the kidney, for associations with uric acid and gout in a Chinese population.

## Materials and Methods

### Experimental Design

A total of 483 gout patients who were all clinically diagnosed according to the American College of Rheumatology diagnostic criteria [12] were enrolled from Huashan Hospital, Changhai Hospital, Taizhou People’s Hospital, Taixing People’s Hospital and Renji Hospital. A total of 835 individuals without a history of gout were enrolled to test the association between the above loci and uric acid. The association with uric acid was tested by linear regression adjusted for gender and age. Fisher's exact test was used to test the association between the loci and gout. A body mass index (BMI) subgroup was used in the uric acid analysis.

### Participants

This study was approved by the Ethical Committees of the School of Life Sciences of Fudan University (approval number of 140). All participants provided written informed consent to participate in this study; 483 gout cases were enrolled, and 835 individuals without a history of gout were enrolled from Taizhou Longitudinal Study [13]. The above 835 individuals were divided into subgroups of normal and overweight according to body mass index (BMI) values following the categories of the World Health Organization (WHO) [14]. Of them, 389 individuals with normal serum urate (≤ 7 mg/dl) were treated as healthy controls [15] in a case-control study for gout. The characteristics of the individuals in this study are shown in S1 Table.

### Genetic analysis

Peripheral blood was collected from all the individuals investigated in our study. Genomic DNA was isolated from whole blood using the QIAamp DNA Blood Mini kit (QIAGEN, Germany) and stored at -20℃. The DNA concentration and quality (including optical density (OD) 260/280 and 260/230 measurements) were determined using a Nanodrop Lite spectrophotometer (Thermo Fisher Scientific, Waltham, MA, USA). Genotyping of rs2544390 in the *LRP2* gene and rs4680 in the *COMT* gene was performed by SNaPshot.

### Statistical analysis

Genotype data of the loci were checked for deviation from Hardy—Weinberg equilibrium. Associations with uric acid were tested by linear regression, adjusting for gender and age. Fisher's exact test was used to test the association between each locus and gout, and the body mass index (BMI) subgroup was used in the uric acid analysis. *P* values less than 0.025 were considered statistically significant after multiple testing using Bonferroni correction.

A meta-analysis was used to test the association between rs2544390 and gout. Two published studies were selected from the PubMed database following predefined criteria. The Q-statistic was calculated to determine the heterogeneity between studies: when heterogeneity was *P*_Het_ < 0.05, a fixed-effect model was used; otherwise, a random-effect model was utilized.

All statistical analyses were performed using STATA statistical software (Version 12.0; StataCorp, College Station, TX, USA) and R (Version 3.0.2: www.r-project.org/).

## Results

### Association of *LRP2* and *COMT* variants with uric acid

Linear regression adjusted for gender and age was performed to test the association between *LRP2* and *COMT* gene variants and uric acid. Rs2544390 in *LRP2* was found to have no significant effect on the levels of serum uric acid in this Chinese population (*P* = 0.440) (Table 1), which was consistent with the conclusion for another Chinese population of 3451 individuals [11]. When analyzing the BMI subgroup, a different result for the association of rs2544390 with uric acid was revealed (Table 1). In the normal weight subgroup (18.5 ≦ BMI < 25), no significant correlation was found between rs2544390 and uric acid (*P* = 0.784), whereas in the overweight subgroup (BMI ≧ 25), rs2544390 had a significant effect on the concentration of uric acid (*P* = 0.022). According to these findings, the association between rs2544390 and uric acid might be modified by BMI.

**Table 1:** Association between SNPs and uric acid in BMI subgroup.

				Minor/	Total		18.5 ≦ BMI < 25		BMI ≧ 25	
SNP	Chr	Locus	Number	major allele	Effect size	*P* values	Effect size	*P* values	Effect size	*P* values
*LRP2*										
rs2544390	2	intron	835	A/G	3.855	0.440	-2.911	0.784	21.623	0.022
*COMT*										
rs4680	22	exon	835	T/C	-3.690	0.511	-14.971	0.204	-4.734	0.648

MAF, minor allele frequency; Effect size, the effect of a minor allele on uric acid; Chr, chromosome

*P*-values of SNP were tested by linear regression with adjustment for gender and age

Regarding rs4680, an SNP in a *COMT* gene exon, no significant correlation with uric acid in either total sample (*P* = 0.511) or by BMI subgroup (*P* = 0.204 and 0.648, separately) was found (Table 1).

### Association of *LRP2* and *COMT* variants with gout

The *P* values of Hardy—Weinberg equilibrium for rs2544390 and rs4680 in the controls were 0.884 and 0.793, suggesting no deviation from Hardy—Weinberg equilibrium. The minor allele frequencies (MAFs) for rs2544390 and rs4680 were more than 0.25 in both cases and in the controls, indicating that these SNPs are very common in both groups (Table 2).

**Table 2:** Association analysis of *COMT* and *LRP2* variants with gout.

		Allele								
SNP		Case			Control			OR	95% CI	*P* values
		1	2	MAF	1	2	MAF			
*COMT*										
rs4680	A/G	243	723	0.25	237	541	0.30	0.77	0.62–0.95	0.015
*LRP2*										
rs2544390	T/C	459	507	0.48	326	452	0.42	1.26	1.03–1.53	0.020

MAF, minor allele frequency; OR, odds ratio; CI, confidence interval

The minor allele was considered as allele 1 and major allele as allele 2

*P*-values of the SNPs were tested by Fisher's exact test

**Fig 1:**
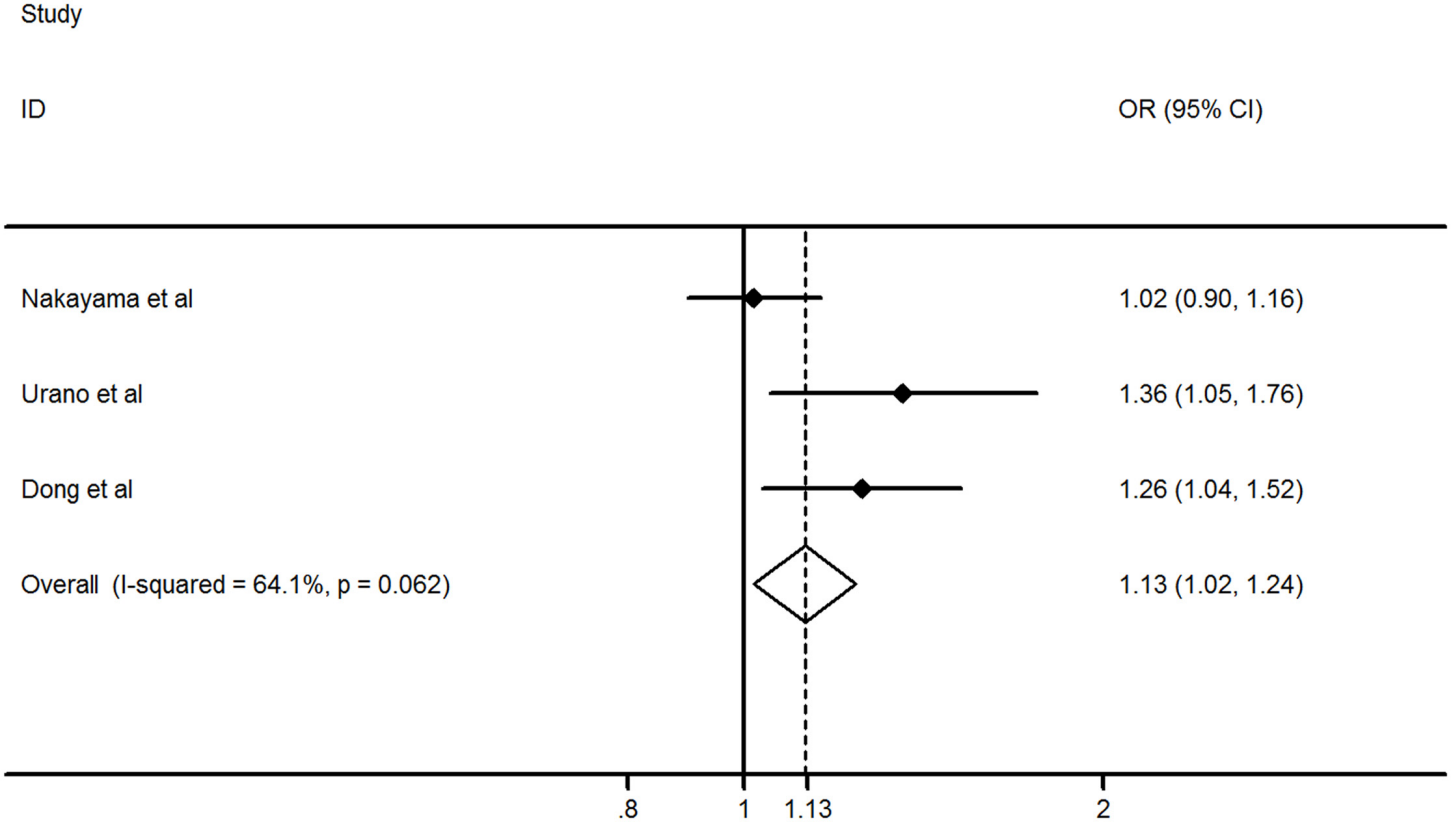
Odds ratios (ORs) and 95% confidence intervals (CI) from each study about the association of the rs2544390 polymorphism and gout in Asians. A meta-analysis in fixed-effect model was used to test the association.

The distributions of T allele frequency in rs2544390 showed a significant difference between the gout patients and healthy controls (0.48 in cases vs 0.42 in controls) in this Chinese population; according to Fisher's exact test, the T allele of rs2544390 exhibited an association with the risk of developing gout (OR = 1.26, *P* = 0.020) (Table 2). To confirm the association in Asians, a meta-analysis pooled the results of this study and two previous studies in Japanese populations with inconsistent results [16, 17]. The results demonstrated a significant association between rs2544390 and gout, with the T allele being a risk factor for the development of gout in Asians (OR = 1.13, *P* = 0.019) (Fig 1).

The distributions of A allele frequency for rs4680 were significantly different between the gout patients and healthy controls (0.25 in cases vs 0.30 in controls), though a missense variation in rs4680 (G > A) decreased the risk of gout (OR = 0.77, *P* = 0.015) (Table 2). To the best of our knowledge, this is the first report of the significant association between *COMT* and the development of gout.

## Discussion

Hyperuricemia is a key risk factor for the development of gout. Urate is synthesized mainly in the liver, and approximately two-thirds of daily urate excretion occurs via the kidneys [1, 2]. A poor renal urate disposal ability leads to nearly 90% of gout cases [3]. In a previous GWAS study, 28 loci were identified as associated with uric acid [5]. However, only 7% of the variance in serum uric acid concentrations can be explained by those loci, and only a portion of the loci were reported to be associated with gout [5, 6]. Therefore, it is necessary to identify novel candidate loci, such as *COMT*, for associations with uric acid and gout.

Catechol-O-methyltransferase (COMT) [7, 8] is expressed ubiquitously and regulates renal dopamine activity. The highest COMT activity occurs in the liver and kidney, and the notable expression of COMT in kidney proximal tubular epithelial cells is considered to indirectly regulate the metabolism of dopamine and other catecholamines [18]. Rs4680 is a common missense variant in the *COMT* gene, and a single-nucleotide substitution from G to A results in an amino acid change from valine (Val) to methionine (Met) at residue 158. Three genotype groups (homozygous Val158Val and Met158Met, heterozygous Val158Met) result from this substitution, leading to different enzyme activities based on changes in thermostability [19]. The Met/Met genotype results in a 3~4-fold reduction in enzymatic activity compared to the Val/Val genotype [20] and leads to higher dopamine levels with the lower rate of catabolism. Dopamine has an effect on several renal functions, such as glomerular filtration and sodium excretion [7]. Furthermore, dopamine-induced glomerular filtration has been reported to be negatively correlated with uric acid concentrations and urate excretion [9]. In this study, we revealed that rs4680G>A plays a protective role in the development of gout, suggesting that rs4680 in *COMT* might influence the development of gout by regulating dopamine levels.

LRP2, a member of the low-density lipoprotein receptor (LDLR) family expressed on the apical surface of epithelial cells [21], is an endocytic receptor that binds and internalizes a variety of ligands, such as lipoproteins, nutrients, morphogens, hormones and their carrier proteins, protease-protease inhibitor complexes, and vitamin-vitamin binding protein complexes [21, 22]. A recent GWAS in a Japanese population suggested an association between urate and rs2544390, a common variant in *LRP2* [10]; in contrast, another GWAS in a Chinese population showed no significant association between rs2544390 and uric acid levels [11]. In the present study, we also found that rs2543390 had no significant influence on uric acid. However, when analyzing by subgroup, rs2544390 showed a significant effect on uric acid concentrations in the overweight subgroup. Another study with 5016 individuals also showed different results among BMI subgroups [23]. In addition, BMI has been proven to be an important factor influencing levels of uric acid. Based on these results, the association between rs2544390 and uric acid appear to be modified by BMI, and BMI might be a heterogeneity factor resulting in the observed discrepant results.

The population-specific effects of *LRP2* in gout have been proven [6]; for instance, the T allele of rs2544390 was found to be associated with increased risk of gout in a combined Māori and Pacific Island cohort, whereas a protective effect was found in European subjects [24]. In addition, inconsistent results have been presented in two reports in Asians. Therefore, it was necessary to study the association in Chinese populations, which have not been previously tested. In our study, the SNP rs2544390 in *LRP2* was found to be associated with the development of gout in the Chinese population, a result that was further confirmed in Asians by a meta-analysis from three studies in Chinese and Japanese populations.

However, there were several limitations in this study. First, a limited sample size was used in this study. Second, some environmental factors associated with uric acid and gout were not assessed. Therefore, further studies of *LRP2* and *COMT* genes in larger samples will be performed to confirm the relationship between these loci and gout together with gene-environment interactions.

In this study, we, for the first time, demonstrate that a common missense variant of *COMT*, rs4680, plays a protective role in the pathogenesis of gout. In addition, we show that rs2544390, an *LRP2* SNP, only impacts uric acid levels in individuals with BMI ≥ 25, which might be an explanation for the controversial results among previous studies. Furthermore, we first identified the association between *LRP2* and gout in a Chinese population and confirm the association in Asians.

## Supporting Information

S1 Table Characteristics of participants according to BMI*. * All data were collected by general health questionnaire, baseline measurements and physical examination.
(DOCX)

S2 Table Genotype and characteristics of each study subject. (DOCX)
