# Supplementary material for: Common Variants in LRP2 and COMT Genes Affect the Susceptibility of Gout in a Chinese Population
Source: PLoS One. 2015 Jul 6;10(7):e0131302. doi: 10.1371/journal.pone.0131302 (PMC4493088; doi:10.1371/journal.pone.0131302)
Supplement: S1 Table — * All data were collected by general health questionnaire, baseline measurements and physical examination. (DOCX) [file pone.0131302.s001.docx]

**S1 Table. Characteristics of participants according to BMI*.**

| Characteristics | 18.5 ≦ BMI ﹤ 25 | BMI ≧ 25 |
| --- | --- | --- |
| Number | 230 | 237 |
| Male (%) | 66.5 | 68.8 |
| Age^#^ | 68.1 (9.0) | 67.5 (6.5) |
| Uric Acid^#^ (umol/L) | 361.5 (117.0) | 388.6 (107.5) |
| BMI^#^ | 22.8 (1.6) | 27.9 (2.4) |
| Smoker (%) | 73.5 | 67.5 |

***** All data were collected by general health questionnaire, baseline measurements and physical examination.

^#^Data of age, uric acid and body mass index (BMI) are expressed as mean (SD).
