# Supplementary material for: Common Variants in LRP2 and COMT Genes Affect the Susceptibility of Gout in a Chinese Population
Source: PLoS One. 2015 Jul 6;10(7):e0131302. doi: 10.1371/journal.pone.0131302 (PMC4493088; doi:10.1371/journal.pone.0131302)
Supplement: S2 Table — (DOCX) [file pone.0131302.s002.docx]

**S2 Table. Genotype and characteristic of each study subject.**

| Sample | Type | Gender | age | rs2544390 | rs4680 | UA |
| --- | --- | --- | --- | --- | --- | --- |
| 15000001 | Non-gout | Female | 66 | CC | AG | 235 |
| 15000002 | Non-gout | Female | 65 | CC | GG | 462 |
| 15000003 | Non-gout | Male | 66 | CC | GG | 436 |
| 15000004 | Non-gout | Female | 57 | CC | AG | 248 |
| 15000005 | Non-gout | Male | 65 | CC | AG | 431 |
| 15000006 | Non-gout | Male | 74 | CC | GG | 586 |
| 15000007 | Non-gout | Female | 59 | CC | AG | 206 |
| 15000008 | Non-gout | Female | 58 | CC | GG | 272 |
| 15000009 | Non-gout | Female | 66 | CC | AG | 211 |
| 15000010 | Non-gout | Male | 70 | CC | AG | 432 |
| 15000011 | Non-gout | Female | 63 | CC | AG | 279 |
| 15000012 | Non-gout | Female | 60 | CC | GG | 235 |
| 15000013 | Non-gout | Male | 60 | CC | GG | 445 |
| 15000014 | Non-gout | Male | 68 | CC | GG | 541 |
| 15000015 | Non-gout | Female | 60 | CC | AG | 429 |
| 15000016 | Non-gout | Female | 65 | CC | AG | 288 |
| 15000017 | Non-gout | Female | 64 | CC | AG | 200 |
| 15000018 | Non-gout | Female | 62 | CC | AG | 207 |
| 15000019 | Non-gout | Male | 78 | CC | GG | 199 |
| 15000020 | Non-gout | Male | 74 | CC | GG | 324 |
| 15000021 | Non-gout | Female | 65 | CC | GG | 423 |
| 15000022 | Non-gout | Male | 65 | CC | AA | 426 |
| 15000023 | Non-gout | Male | 67 | CC | GG | 367 |
| 15000024 | Non-gout | Male | 68 | CC | GG | 230 |
| 15000025 | Non-gout | Male | 61 | CC | AA | 340 |
| 15000026 | Non-gout | Male | 67 | CC | AG | 623 |
| 15000027 | Non-gout | Female | 78 | CC | GG | 439 |
| 15000028 | Non-gout | Male | 69 | CC | AA | 376 |
| 15000029 | Non-gout | Male | 68 | CC | GG | 274 |
| 15000030 | Non-gout | Male | 68 | CC | GG | 433 |
| 15000031 | Non-gout | Male | 73 | CC | AG | 429 |
| 15000032 | Non-gout | Female | 59 | CC | GG | 300 |
| 15000033 | Non-gout | Male | 66 | CC | AA | 366 |
| 15000034 | Non-gout | Male | 71 | CC | GG | 233 |
| 15000035 | Non-gout | Male | 65 | CC | GG | 621 |
| 15000036 | Non-gout | Male | 60 | CC | GG | 286 |
| 15000037 | Non-gout | Female | 66 | CC | GG | 488 |
| 15000038 | Non-gout | Male | 66 | CC | AG | 451 |
| 15000039 | Non-gout | Female | 63 | CC | AG | 223 |
| 15000040 | Non-gout | Female | 62 | CC | GG | 451 |
| 15000041 | Non-gout | Male | 61 | CC | AG | 422 |
| 15000042 | Non-gout | Male | 65 | CC | AG | 299 |
| 15000043 | Non-gout | Male | 65 | CC | AG | 428 |
| 15000044 | Non-gout | Male | 64 | CC | AA | 412 |
| 15000045 | Non-gout | Male | 65 | CC | AG | 472 |
| 15000046 | Non-gout | Male | 82 | CC | AG | 413 |
| 15000047 | Non-gout | Male | 71 | CC | GG | 667 |
| 15000048 | Non-gout | Male | 67 | CC | GG | 361 |
| 15000049 | Non-gout | Male | 72 | CC | AG | 197 |
| 15000050 | Non-gout | Female | 63 | CC | AG | 282 |
| 15000051 | Non-gout | Female | 64 | CC | GG | 427 |
| 15000052 | Non-gout | Male | 65 | CC | AG | 426 |
| 15000053 | Non-gout | Male | 61 | CC | GG | 411 |
| 15000054 | Non-gout | Male | 72 | CC | AG | 422 |
| 15000055 | Non-gout | Male | 65 | CC | AG | 309 |
| 15000056 | Non-gout | Male | 82 | CC | GG | 378 |
| 15000057 | Non-gout | Male | 68 | CC | GG | 323 |
| 15000058 | Non-gout | Male | 68 | CC | AG | 275 |
| 15000059 | Non-gout | Male | 74 | CC | AG | 305 |
| 15000060 | Non-gout | Male | 62 | CC | AG | 440 |
| 15000061 | Non-gout | Male | 50 | CC | AG | 217 |
| 15000062 | Non-gout | Male | 69 | CC | GG | 412 |
| 15000063 | Non-gout | Female | 67 | CC | GG | 234 |
| 15000064 | Non-gout | Female | 71 | CC | GG | 427 |
| 15000065 | Non-gout | Male | 73 | CC | AG | 545 |
| 15000066 | Non-gout | Male | 71 | CC | GG | 276 |
| 15000067 | Non-gout | Male | 64 | CC | AG | 438 |
| 15000068 | Non-gout | Male | 70 | CC | GG | 291 |
| 15000069 | Non-gout | Male | 70 | CC | GG | 438 |
| 15000070 | Non-gout | Male | 69 | CC | GG | 427 |
| 15000071 | Non-gout | Female | 62 | CC | AG | 239 |
| 15000072 | Non-gout | Male | 69 | CC | GG | 451 |
| 15000073 | Non-gout | Male | 63 | CC | AG | 383 |
| 15000074 | Non-gout | Male | 92 | CC | GG | 430 |
| 15000075 | Non-gout | Male | 66 | CC | AA | 259 |
| 15000076 | Non-gout | Male | 75 | CC | AG | 454 |
| 15000077 | Non-gout | Male | 73 | CC | GG | 447 |
| 15000078 | Non-gout | Female | 80 | CC | AG | 599 |
| 15000079 | Non-gout | Male | 75 | CC | AA | 294 |
| 15000080 | Non-gout | Male | 70 | CC | GG | 392 |
| 15000081 | Non-gout | Male | 69 | CC | GG | 294 |
| 15000082 | Non-gout | Male | 68 | CC | AG | 472 |
| 15000083 | Non-gout | Female | 67 | CC | AG | 334 |
| 15000084 | Non-gout | Male | 68 | CC | AA | 233 |
| 15000085 | Non-gout | Male | 73 | CC | AG | 427 |
| 15000086 | Non-gout | Male | 74 | CC | AG | 310 |
| 15000087 | Non-gout | Male | 72 | CC | GG | 308 |
| 15000088 | Non-gout | Male | 73 | CC | GG | 323 |
| 15000089 | Non-gout | Male | 114 | CC | GG | 436 |
| 15000090 | Non-gout | Male | 60 | CC | GG | 432 |
| 15000091 | Non-gout | Male | 77 | CC | GG | 493 |
| 15000092 | Non-gout | Female | 62 | CC | AG | 278 |
| 15000093 | Non-gout | Female | 60 | CC | GG | 224 |
| 15000094 | Non-gout | Male | 68 | CC | AG | 479 |
| 15000095 | Non-gout | Male | 65 | CC | GG | 518 |
| 15000096 | Non-gout | Female | 54 | CC | GG | 191 |
| 15000097 | Non-gout | Female | 64 | CC | AG | 231 |
| 15000098 | Non-gout | Female | 66 | CC | GG | 477 |
| 15000099 | Non-gout | Male | 84 | CC | AG | 327 |
| 15000100 | Non-gout | Male | 68 | CC | GG | 433 |
| 15000101 | Non-gout | Male | 85 | CC | GG | 416 |
| 15000102 | Non-gout | Male | 51 | CC | AG | 454 |
| 15000103 | Non-gout | Female | 63 | CC | AA | 241 |
| 15000104 | Non-gout | Female | 58 | CC | GG | 444 |
| 15000105 | Non-gout | Male | 71 | CC | AG | 291 |
| 15000106 | Non-gout | Female | 56 | CC | GG | 265 |
| 15000107 | Non-gout | Male | 61 | CC | AG | 485 |
| 15000108 | Non-gout | Female | 61 | CC | AG | 360 |
| 15000109 | Non-gout | Female | 63 | CC | GG | 263 |
| 15000110 | Non-gout | Male | 62 | CC | AA | 326 |
| 15000111 | Non-gout | Female | 62 | CC | GG | 237 |
| 15000112 | Non-gout | Female | 79 | CC | AA | 477 |
| 15000113 | Non-gout | Male | 62 | CC | GG | 438 |
| 15000114 | Non-gout | Male | 65 | CC | GG | 442 |
| 15000115 | Non-gout | Female | 79 | CC | GG | 435 |
| 15000116 | Non-gout | Male | 67 | CC | GG | 273 |
| 15000117 | Non-gout | Male | 60 | CC | GG | 585 |
| 15000118 | Non-gout | Male | 66 | CC | AG | 482 |
| 15000119 | Non-gout | Male | 81 | CC | GG | 427 |
| 15000120 | Non-gout | Male | 67 | CC | AG | 239 |
| 15000121 | Non-gout | Male | 68 | CC | GG | 342 |
| 15000122 | Non-gout | Female | 62 | CC | AG | 226 |
| 15000123 | Non-gout | Male | 71 | CC | GG | 457 |
| 15000124 | Non-gout | Male | 62 | CC | AG | 524 |
| 15000125 | Non-gout | Male | 63 | CC | AG | 283 |
| 15000126 | Non-gout | Male | 70 | CC | AG | 300 |
| 15000127 | Non-gout | Female | 65 | CC | AG | 412 |
| 15000128 | Non-gout | Male | 70 | CC | AG | 254 |
| 15000129 | Non-gout | Female | 63 | CC | GG | 635 |
| 15000130 | Non-gout | Male | 72 | CC | GG | 262 |
| 15000131 | Non-gout | Female | 59 | CC | AG | 252 |
| 15000132 | Non-gout | Female | 65 | CC | GG | 177 |
| 15000133 | Non-gout | Male | 65 | CC | GG | 455 |
| 15000134 | Non-gout | Female | 59 | CC | AG | 247 |
| 15000135 | Non-gout | Female | 60 | CC | GG | 300 |
| 15000136 | Non-gout | Female | 60 | CC | GG | 453 |
| 15000137 | Non-gout | Male | 94 | CC | AG | 542 |
| 15000138 | Non-gout | Male | 81 | CC | GG | 215 |
| 15000139 | Non-gout | Female | 58 | CC | AG | 233 |
| 15000140 | Non-gout | Female | 56 | CC | AA | 247 |
| 15000141 | Non-gout | Male | 85 | CC | AG | 399 |
| 15000142 | Non-gout | Male | 62 | CC | GG | 249 |
| 15000143 | Non-gout | Male | 80 | CC | GG | 354 |
| 15000144 | Non-gout | Female | 55 | CC | AA | 515 |
| 15000145 | Non-gout | Male | 56 | CC | AG | 286 |
| 15000146 | Non-gout | Male | 71 | CC | GG | 682 |
| 15000147 | Non-gout | Female | 70 | CC | GG | 435 |
| 15000148 | Non-gout | Male | 70 | CC | AG | 318 |
| 15000149 | Non-gout | Male | 65 | CC | AA | 602 |
| 15000150 | Non-gout | Female | 68 | CC | AA | 598 |
| 15000151 | Non-gout | Male | 63 | CC | AA | 597 |
| 15000152 | Non-gout | Female | 66 | CC | AG | 594 |
| 15000153 | Non-gout | Male | 48 | CC | AG | 581 |
| 15000154 | Non-gout | Male | 72 | CC | GG | 580 |
| 15000155 | Non-gout | Male | 76 | CC | GG | 577 |
| 15000156 | Non-gout | Female | 81 | CC | GG | 576 |
| 15000157 | Non-gout | Male | 82 | CC | AG | 566 |
| 15000158 | Non-gout | Male | 73 | CC | GG | 560 |
| 15000159 | Non-gout | Male | 82 | CC | GG | 557 |
| 15000160 | Non-gout | Male | 37 | CC | AA | 546 |
| 15000161 | Non-gout | Male | 24 | CC | AG | 537 |
| 15000162 | Non-gout | Male | 78 | CC | GG | 534 |
| 15000163 | Non-gout | Male | 66 | CC | GG | 533 |
| 15000164 | Non-gout | Male | 84 | CC | GG | 531 |
| 15000165 | Non-gout | Male | 72 | CC | GG | 523 |
| 15000166 | Non-gout | Male | 70 | CC | GG | 523 |
| 15000167 | Non-gout | Male | 64 | CC | GG | 515 |
| 15000168 | Non-gout | Male | 64 | CC | GG | 512 |
| 15000169 | Non-gout | Male | 67 | CC | GG | 505 |
| 15000170 | Non-gout | Male | 36 | CC | GG | 504 |
| 15000171 | Non-gout | Male | 69 | CC | GG | 496 |
| 15000172 | Non-gout | Male | 70 | CC | GG | 493 |
| 15000173 | Non-gout | Male | 28 | CC | AG | 491 |
| 15000174 | Non-gout | Male | 54 | CC | GG | 488 |
| 15000175 | Non-gout | Female | 67 | CC | GG | 483 |
| 15000176 | Non-gout | Male | 62 | CC | GG | 482 |
| 15000177 | Non-gout | Female | 70 | CC | GG | 482 |
| 15000178 | Non-gout | Male | 81 | CC | AA | 481 |
| 15000179 | Non-gout | Male | 84 | CC | AG | 479 |
| 15000180 | Non-gout | Male | 39 | CC | AA | 478 |
| 15000181 | Non-gout | Male | 76 | CC | AG | 476 |
| 15000182 | Non-gout | Male | 63 | CC | AG | 475 |
| 15000183 | Non-gout | Male | 66 | CC | AA | 473 |
| 15000184 | Non-gout | Male | 82 | CC | GG | 473 |
| 15000185 | Non-gout | Male | 76 | CC | GG | 472 |
| 15000186 | Non-gout | Male | 70 | CC | GG | 471 |
| 15000187 | Non-gout | Female | 79 | CC | GG | 470 |
| 15000188 | Non-gout | Female | 87 | CC | GG | 466 |
| 15000189 | Non-gout | Male | 64 | CC | GG | 465 |
| 15000190 | Non-gout | Male | 83 | CC | AG | 465 |
| 15000191 | Non-gout | Male | 77 | CC | GG | 464 |
| 15000192 | Non-gout | Male | 86 | CC | AG | 459 |
| 15000193 | Non-gout | Female | 77 | CC | GG | 455 |
| 15000194 | Non-gout | Male | 80 | CC | GG | 453 |
| 15000195 | Non-gout | Female | 62 | CC | AA | 450 |
| 15000196 | Non-gout | Male | 76 | CC | AG | 444 |
| 15000197 | Non-gout | Male | 89 | CC | GG | 444 |
| 15000198 | Non-gout | Male | 63 | CC | GG | 443 |
| 15000199 | Non-gout | Female | 76 | CC | AG | 443 |
| 15000200 | Non-gout | Male | 52 | CC | AG | 443 |
| 15000201 | Non-gout | Male | 58 | CC | GG | 441 |
| 15000202 | Non-gout | Male | 74 | CC | AG | 438 |
| 15000203 | Non-gout | Male | 88 | CC | AG | 438 |
| 15000204 | Non-gout | Male | 64 | CC | GG | 438 |
| 15000205 | Non-gout | Male | 81 | CC | GG | 435 |
| 15000206 | Non-gout | Male | 73 | CC | GG | 435 |
| 15000207 | Non-gout | Female | 75 | CC | AG | 434 |
| 15000208 | Non-gout | Female | 81 | CC | AA | 433 |
| 15000209 | Non-gout | Male | 75 | CC | GG | 432 |
| 15000210 | Non-gout | Female | 81 | CC | GG | 432 |
| 15000211 | Non-gout | Male | 78 | CC | AG | 432 |
| 15000212 | Non-gout | Male | 78 | CC | AG | 431 |
| 15000213 | Non-gout | Male | 80 | CC | GG | 431 |
| 15000214 | Non-gout | Female | 81 | CC | GG | 430 |
| 15000215 | Non-gout | Female | 67 | CC | AG | 428 |
| 15000216 | Non-gout | Male | 64 | CC | AA | 428 |
| 15000217 | Non-gout | Male | 73 | CC | GG | 425 |
| 15000218 | Non-gout | Female | 69 | CC | GG | 424 |
| 15000219 | Non-gout | Male | 73 | CC | AG | 423 |
| 15000220 | Non-gout | Female | 76 | CC | AG | 421 |
| 15000221 | Non-gout | Male | 35 | CC | AG | 420 |
| 15000222 | Non-gout | Male | 62 | CC | GG | 415 |
| 15000223 | Non-gout | Male | 49 | CC | GG | 398 |
| 15000224 | Non-gout | Male | 62 | CC | AA | 376 |
| 15000225 | Non-gout | Male | 39 | CC | AG | 374 |
| 15000226 | Non-gout | Male | 36 | CC | GG | 372 |
| 15000227 | Non-gout | Male | 46 | CC | GG | 370 |
| 15000228 | Non-gout | Male | 40 | CC | AA | 360 |
| 15000229 | Non-gout | Male | 73 | CC | GG | 358 |
| 15000230 | Non-gout | Male | 44 | CC | AG | 356 |
| 15000231 | Non-gout | Female | 36 | CC | AG | 356 |
| 15000232 | Non-gout | Male | 47 | CC | AA | 355 |
| 15000233 | Non-gout | Male | 34 | CC | GG | 349 |
| 15000234 | Non-gout | Male | 29 | CC | GG | 349 |
| 15000235 | Non-gout | Male | 48 | CC | AG | 349 |
| 15000236 | Non-gout | Female |  | CC | GG | 347 |
| 15000237 | Non-gout | Male |  | CC | AG | 345 |
| 15000238 | Non-gout | Male | 35 | CC | AG | 344 |
| 15000239 | Non-gout | Male | 49 | CC | AG | 344 |
| 15000240 | Non-gout | Male | 44 | CC | AG | 341 |
| 15000241 | Non-gout | Female | 57 | CC | AG | 340 |
| 15000242 | Non-gout | Male | 46 | CC | AG | 329 |
| 15000243 | Non-gout | Female | 51 | CC | GG | 328 |
| 15000244 | Non-gout | Male | 33 | CC | GG | 328 |
| 15000245 | Non-gout | Male | 63 | CC | GG | 326 |
| 15000246 | Non-gout | Male | 34 | CC | AG | 316 |
| 15000247 | Non-gout | Male | 62 | CC | GG | 310 |
| 15000248 | Non-gout | Female | 58 | CC | AG | 309 |
| 15000249 | Non-gout | Male | 39 | CC | AG | 308 |
| 15000250 | Non-gout | Female | 38 | CC | GG | 308 |
| 15000251 | Non-gout | Female | 59 | CC | AG | 299 |
| 15000252 | Non-gout | Female | 43 | CC | GG | 294 |
| 15000253 | Non-gout | Male | 80 | CC | AG | 291 |
| 15000254 | Non-gout | Female | 37 | CC | AG | 287 |
| 15000255 | Non-gout | Male | 48 | CC | AG | 280 |
| 15000256 | Non-gout | Male | 32 | CC | GG | 279 |
| 15000257 | Non-gout | Female |  | CC | AG | 279 |
| 15000258 | Non-gout | Female |  | CC | AA | 279 |
| 15000259 | Non-gout | Male | 39 | CC | GG | 277 |
| 15000260 | Non-gout | Female | 34 | CC | GG | 275 |
| 15000261 | Non-gout | Male | 67 | CC | GG | 269 |
| 15000262 | Non-gout | Female | 21 | CC | AG | 266 |
| 15000263 | Non-gout | Female | 33 | CC | GG | 261 |
| 15000264 | Non-gout | Female | 58 | CC | AG | 259 |
| 15000265 | Non-gout | Female | 31 | CC | GG | 247 |
| 15000266 | Non-gout | Male | 48 | CC | GG | 243 |
| 15000267 | Non-gout | Male | 51 | CC | GG | 224 |
| 15000268 | Non-gout | Female | 36 | CC | GG | 201 |
| 15000269 | Non-gout |  |  | CC | GG | 162 |
| 15000270 | Non-gout | Female | 64 | TC | AG | 471 |
| 15000271 | Non-gout | Male | 77 | TC | GG | 468 |
| 15000272 | Non-gout | Male | 68 | TC | GG | 465 |
| 15000273 | Non-gout | Female | 78 | TC | AG | 467 |
| 15000274 | Non-gout | Male | 59 | TC | AG | 433 |
| 15000275 | Non-gout | Male | 68 | TC | GG | 403 |
| 15000276 | Non-gout | Female | 61 | TC | GG | 225 |
| 15000277 | Non-gout | Male | 68 | TC | GG | 469 |
| 15000278 | Non-gout | Female | 65 | TC | GG | 534 |
| 15000279 | Non-gout | Female | 63 | TC | GG | 216 |
| 15000280 | Non-gout | Male | 84 | TC | AG | 255 |
| 15000281 | Non-gout | Female | 66 | TC | AG | 441 |
| 15000282 | Non-gout | Female | 57 | TC | GG | 285 |
| 15000283 | Non-gout | Female | 70 | TC | GG | 452 |
| 15000284 | Non-gout | Male | 63 | TC | GG | 424 |
| 15000285 | Non-gout | Female | 58 | TC | GG | 435 |
| 15000286 | Non-gout | Male | 71 | TC | GG | 397 |
| 15000287 | Non-gout | Male | 80 | TC | GG | 533 |
| 15000288 | Non-gout | Female | 78 | TC | AG | 468 |
| 15000289 | Non-gout | Male | 63 | TC | GG | 395 |
| 15000290 | Non-gout | Female | 60 | TC | AA | 162 |
| 15000291 | Non-gout | Male | 70 | TC | GG | 328 |
| 15000292 | Non-gout | Male | 65 | TC | GG | 270 |
| 15000293 | Non-gout | Male | 65 | TC | AG | 333 |
| 15000294 | Non-gout | Male | 68 | TC | AG | 412 |
| 15000295 | Non-gout | Male | 67 | TC | GG | 232 |
| 15000296 | Non-gout | Male | 67 | TC | AG | 438 |
| 15000297 | Non-gout | Male | 67 | TC | AG | 362 |
| 15000298 | Non-gout | Male | 71 | TC | AG | 478 |
| 15000299 | Non-gout | Female | 64 | TC | AG | 390 |
| 15000300 | Non-gout | Male | 55 | TC | AA | 490 |
| 15000301 | Non-gout | Female | 68 | TC | GG | 463 |
| 15000302 | Non-gout | Male | 71 | TC | AG | 494 |
| 15000303 | Non-gout | Male | 72 | TC | GG | 538 |
| 15000304 | Non-gout | Male | 67 | TC | GG | 489 |
| 15000305 | Non-gout | Female | 62 | TC | GG | 449 |
| 15000306 | Non-gout | Male | 74 | TC | GG | 542 |
| 15000307 | Non-gout | Male | 55 | TC | GG | 486 |
| 15000308 | Non-gout | Female | 66 | TC | AG | 346 |
| 15000309 | Non-gout | Female | 66 | TC | GG | 311 |
| 15000310 | Non-gout | Male | 70 | TC | AG | 339 |
| 15000311 | Non-gout | Male | 68 | TC | AG | 454 |
| 15000312 | Non-gout | Male | 74 | TC | AG | 437 |
| 15000313 | Non-gout | Male | 64 | TC | GG | 518 |
| 15000314 | Non-gout | Male | 72 | TC | AG | 479 |
| 15000315 | Non-gout | Male | 56 | TC | AG | 491 |
| 15000316 | Non-gout | Male | 84 | TC | AG | 446 |
| 15000317 | Non-gout | Male | 71 | TC | GG | 468 |
| 15000318 | Non-gout | Male | 76 | TC | GG | 427 |
| 15000319 | Non-gout | Female | 60 | TC | AG | 283 |
| 15000320 | Non-gout | Male | 66 | TC | GG | 459 |
| 15000321 | Non-gout | Male | 73 | TC | AG | 522 |
| 15000322 | Non-gout | Female | 62 | TC | AG | 302 |
| 15000323 | Non-gout | Female | 69 | TC | GG | 225 |
| 15000324 | Non-gout | Male | 64 | TC | AG | 329 |
| 15000325 | Non-gout | Male | 77 | TC | GG | 455 |
| 15000326 | Non-gout | Female | 60 | TC | GG | 227 |
| 15000327 | Non-gout | Male | 73 | TC | GG | 452 |
| 15000328 | Non-gout | Male | 63 | TC | GG | 514 |
| 15000329 | Non-gout | Male | 73 | TC | GG | 376 |
| 15000330 | Non-gout | Male | 82 | TC | GG | 433 |
| 15000331 | Non-gout | Male | 72 | TC | AG | 332 |
| 15000332 | Non-gout | Female | 63 | TC | AA | 183 |
| 15000333 | Non-gout | Male | 66 | TC | GG | 376 |
| 15000334 | Non-gout | Male | 65 | TC | GG | 499 |
| 15000335 | Non-gout | Female | 59 | TC | AA | 237 |
| 15000336 | Non-gout | Male | 62 | TC | AG | 444 |
| 15000337 | Non-gout | Male | 71 | TC | AG | 305 |
| 15000338 | Non-gout | Male | 78 | TC | GG | 426 |
| 15000339 | Non-gout | Male | 66 | TC | AA | 342 |
| 15000340 | Non-gout | Male | 66 | TC | AG | 493 |
| 15000341 | Non-gout | Male | 68 | TC | AA | 458 |
| 15000342 | Non-gout | Female | 73 | TC | AG | 428 |
| 15000343 | Non-gout | Male | 74 | TC | GG | 471 |
| 15000344 | Non-gout | Female | 64 | TC | GG | 339 |
| 15000345 | Non-gout | Female | 69 | TC | AG | 445 |
| 15000346 | Non-gout | Male | 69 | TC | AG | 535 |
| 15000347 | Non-gout | Female | 66 | TC | GG | 181 |
| 15000348 | Non-gout | Male | 72 | TC | AG | 328 |
| 15000349 | Non-gout | Male | 69 | TC | GG | 458 |
| 15000350 | Non-gout | Male | 64 | TC | AG | 294 |
| 15000351 | Non-gout | Female | 56 | TC | GG | 465 |
| 15000352 | Non-gout | Female | 69 | TC | GG | 429 |
| 15000353 | Non-gout | Female | 57 | TC | AG | 446 |
| 15000354 | Non-gout | Female | 60 | TC | AG | 168 |
| 15000355 | Non-gout | Male | 68 | TC | GG | 271 |
| 15000356 | Non-gout | Male | 75 | TC | GG | 483 |
| 15000357 | Non-gout | Female | 73 | TC | GG | 451 |
| 15000358 | Non-gout | Female | 61 | TC | GG | 303 |
| 15000359 | Non-gout | Male | 65 | TC | AG | 388 |
| 15000360 | Non-gout | Male | 63 | TC | AG | 374 |
| 15000361 | Non-gout | Female | 57 | TC | GG | 420 |
| 15000362 | Non-gout | Male | 69 | TC | AG | 381 |
| 15000363 | Non-gout | Female | 56 | TC | GG | 163 |
| 15000364 | Non-gout | Male | 72 | TC | AG | 296 |
| 15000365 | Non-gout | Male | 57 | TC | GG | 440 |
| 15000366 | Non-gout | Female | 73 | TC | AG | 343 |
| 15000367 | Non-gout | Male | 61 | TC | GG | 310 |
| 15000368 | Non-gout | Male | 80 | TC | AG | 410 |
| 15000369 | Non-gout | Male | 69 | TC | GG | 296 |
| 15000370 | Non-gout | Male | 68 | TC | AG | 215 |
| 15000371 | Non-gout | Male | 74 | TC | AA | 390 |
| 15000372 | Non-gout | Male | 77 | TC | AG | 588 |
| 15000373 | Non-gout | Female | 57 | TC | GG | 297 |
| 15000374 | Non-gout | Female | 58 | TC | GG | 181 |
| 15000375 | Non-gout | Male | 68 | TC | GG | 445 |
| 15000376 | Non-gout | Female | 68 | TC | AG | 213 |
| 15000377 | Non-gout | Male | 72 | TC | AG | 491 |
| 15000378 | Non-gout | Male | 81 | TC | GG | 344 |
| 15000379 | Non-gout | Male | 66 | TC | GG | 247 |
| 15000380 | Non-gout | Male | 73 | TC | GG | 341 |
| 15000381 | Non-gout | Female | 63 | TC | AG | 579 |
| 15000382 | Non-gout | Female | 74 | TC | GG | 526 |
| 15000383 | Non-gout | Male | 72 | TC | GG | 191 |
| 15000384 | Non-gout | Female | 73 | TC | GG | 453 |
| 15000385 | Non-gout | Male | 67 | TC | AG | 471 |
| 15000386 | Non-gout | Female | 60 | TC | GG | 271 |
| 15000387 | Non-gout | Male | 63 | TC | AG | 301 |
| 15000388 | Non-gout | Female | 59 | TC | AG | 219 |
| 15000389 | Non-gout | Male | 62 | TC | AG | 445 |
| 15000390 | Non-gout | Male | 69 | TC | GG | 245 |
| 15000391 | Non-gout | Male | 67 | TC | AG | 461 |
| 15000392 | Non-gout | Female | 80 | TC | AA | 462 |
| 15000393 | Non-gout | Male | 64 | TC | AG | 270 |
| 15000394 | Non-gout | Male | 67 | TC | AG | 261 |
| 15000395 | Non-gout | Female | 62 | TC | AG | 206 |
| 15000396 | Non-gout | Female | 57 | TC | AA | 190 |
| 15000397 | Non-gout | Male | 66 | TC | GG | 368 |
| 15000398 | Non-gout | Male | 67 | TC | AG | 292 |
| 15000399 | Non-gout | Male | 62 | TC | AG | 438 |
| 15000400 | Non-gout | Male | 67 | TC | GG | 217 |
| 15000401 | Non-gout | Female | 63 | TC | AG | 327 |
| 15000402 | Non-gout | Male | 61 | TC | AG | 355 |
| 15000403 | Non-gout | Male | 67 | TC | GG | 303 |
| 15000404 | Non-gout | Female | 58 | TC | GG | 698 |
| 15000405 | Non-gout | Male | 78 | TC | GG | 515 |
| 15000406 | Non-gout | Male | 69 | TC | GG | 310 |
| 15000407 | Non-gout | Male | 67 | TC | GG | 700 |
| 15000408 | Non-gout | Male | 74 | TC | GG | 318 |
| 15000409 | Non-gout | Male | 63 | TC | GG | 428 |
| 15000410 | Non-gout | Male | 65 | TC | GG | 248 |
| 15000411 | Non-gout | Male | 56 | TC | GG | 429 |
| 15000412 | Non-gout | Female | 69 | TC | GG | 444 |
| 15000413 | Non-gout | Male | 68 | TC | GG | 443 |
| 15000414 | Non-gout | Male | 59 | TC | AG | 452 |
| 15000415 | Non-gout | Male | 69 | TC | AG | 289 |
| 15000416 | Non-gout | Female | 61 | TC | AG | 206 |
| 15000417 | Non-gout | Male | 67 | TC | GG | 431 |
| 15000418 | Non-gout | Male | 62 | TC | GG | 287 |
| 15000419 | Non-gout | Male | 67 | TC | AA | 477 |
| 15000420 | Non-gout | Male | 70 | TC | AA | 388 |
| 15000421 | Non-gout | Male | 70 | TC | AG | 347 |
| 15000422 | Non-gout | Male | 64 | TC | AA | 481 |
| 15000423 | Non-gout | Male | 79 | TC | AA | 276 |
| 15000424 | Non-gout | Male | 67 | TC | GG | 322 |
| 15000425 | Non-gout | Female | 69 | TC | GG | 231 |
| 15000426 | Non-gout | Female | 63 | TC | GG | 442 |
| 15000427 | Non-gout | Female | 71 | TC | GG | 431 |
| 15000428 | Non-gout | Male | 67 | TC | AG | 267 |
| 15000429 | Non-gout | Female | 64 | TC | GG | 222 |
| 15000430 | Non-gout | Male | 66 | TC | AG | 233 |
| 15000431 | Non-gout | Female | 60 | TC | GG | 235 |
| 15000432 | Non-gout | Female | 60 | TC | AG | 179 |
| 15000433 | Non-gout | Female | 80 | TC | AG | 495 |
| 15000434 | Non-gout | Female | 60 | TC | GG | 269 |
| 15000435 | Non-gout | Male | 94 | TC | AG | 381 |
| 15000436 | Non-gout | Male | 78 | TC | GG | 437 |
| 15000437 | Non-gout | Male | 71 | TC | AA | 393 |
| 15000438 | Non-gout | Female | 59 | TC | AG | 262 |
| 15000439 | Non-gout | Female | 70 | TC | GG | 437 |
| 15000440 | Non-gout | Male | 71 | TC | GG | 437 |
| 15000441 | Non-gout | Male | 67 | TC | GG | 366 |
| 15000442 | Non-gout | Male | 70 | TC | GG | 357 |
| 15000443 | Non-gout | Male | 69 | TC | AG | 321 |
| 15000444 | Non-gout | Male | 68 | TC | GG | 219 |
| 15000445 | Non-gout | Male | 61 | TC | GG | 280 |
| 15000446 | Non-gout | Male | 84 | TC | GG | 394 |
| 15000447 | Non-gout | Female | 53 | TC | GG | 442 |
| 15000448 | Non-gout | Male | 72 | TC | AG | 443 |
| 15000449 | Non-gout | Female | 75 | TC | GG | 179 |
| 15000450 | Non-gout | Female | 59 | TC | GG | 427 |
| 15000451 | Non-gout | Female | 69 | TC | AG | 174 |
| 15000452 | Non-gout | Male | 70 | TC | AG | 643 |
| 15000453 | Non-gout | Male | 76 | TC | AG | 444 |
| 15000454 | Non-gout | Male | 62 | TC | GG | 249 |
| 15000455 | Non-gout | Female | 61 | TC | GG | 146 |
| 15000456 | Non-gout | Male | 72 | TC | AG | 557 |
| 15000457 | Non-gout | Female | 57 | TC | AG | 182 |
| 15000458 | Non-gout | Male | 77 | TC | GG | 493 |
| 15000459 | Non-gout | Female | 64 | TC | AG | 237 |
| 15000460 | Non-gout | Male | 73 | TC | AA | 277 |
| 15000461 | Non-gout | Male | 72 | TC | AG | 463 |
| 15000462 | Non-gout | Male | 66 | TC | GG | 338 |
| 15000463 | Non-gout | Male | 94 | TC | AG | 372 |
| 15000464 | Non-gout | Male | 66 | TC | GG | 397 |
| 15000465 | Non-gout | Female | 68 | TC | AG | 443 |
| 15000466 | Non-gout | Male | 52 | TC | GG | 526 |
| 15000467 | Non-gout | Male | 73 | TC | GG | 683 |
| 15000468 | Non-gout | Male | 66 | TC | AG | 249 |
| 15000469 | Non-gout | Female | 51 | TC | AA | 178 |
| 15000470 | Non-gout | Male | 64 | TC | GG | 539 |
| 15000471 | Non-gout | Male | 68 | TC | AG | 289 |
| 15000472 | Non-gout | Male | 67 | TC | AG | 316 |
| 15000473 | Non-gout | Male | 72 | TC | AG | 231 |
| 15000474 | Non-gout | Male | 71 | TC | GG | 276 |
| 15000475 | Non-gout | Male | 84 | TC | GG | 219 |
| 15000476 | Non-gout | Male | 76 | TC | GG | 422 |
| 15000477 | Non-gout | Female | 70 | TC | GG | 201 |
| 15000478 | Non-gout | Female | 69 | TC | AA | 493 |
| 15000479 | Non-gout | Male | 63 | TC | AG | 297 |
| 15000480 | Non-gout | Male | 64 | TC | GG | 425 |
| 15000481 | Non-gout | Female | 64 | TC | GG | 169 |
| 15000482 | Non-gout | Male | 67 | TC | GG | 318 |
| 15000483 | Non-gout | Male | 69 | TC | GG | 159 |
| 15000484 | Non-gout | Male | 80 | TC | GG | 246 |
| 15000485 | Non-gout | Male | 93 | TC | GG | 575 |
| 15000486 | Non-gout | Male | 68 | TC | GG | 294 |
| 15000487 | Non-gout | Female | 61 | TC | AG | 236 |
| 15000488 | Non-gout | Male | 70 | TC | GG | 432 |
| 15000489 | Non-gout | Male | 93 | TC | AG | 438 |
| 15000490 | Non-gout | Male | 62 | TC | GG | 298 |
| 15000491 | Non-gout | Male | 64 | TC | GG | 378 |
| 15000492 | Non-gout | Female | 62 | TC | AG | 455 |
| 15000493 | Non-gout | Female | 57 | TC | GG | 215 |
| 15000494 | Non-gout | Male | 82 | TC | AG | 343 |
| 15000495 | Non-gout | Male | 74 | TC | AG | 383 |
| 15000496 | Non-gout | Female | 63 | TC | AG | 149 |
| 15000497 | Non-gout | Male | 82 | TC | GG | 705 |
| 15000498 | Non-gout | Female | 71 | TC | GG | 685 |
| 15000499 | Non-gout | Male | 68 | TC | GG | 668 |
| 15000500 | Non-gout | Female | 71 | TC | GG | 609 |
| 15000501 | Non-gout | Female | 66 | TC | AG | 599 |
| 15000502 | Non-gout | Male | 70 | TC | AA | 592 |
| 15000503 | Non-gout | Male | 63 | TC | GG | 571 |
| 15000504 | Non-gout | Male | 64 | TC | GG | 569 |
| 15000505 | Non-gout | Male | 67 | TC | AG | 567 |
| 15000506 | Non-gout | Female | 86 | TC | AA | 560 |
| 15000507 | Non-gout | Male | 42 | TC | GG | 560 |
| 15000508 | Non-gout | Male | 48 | TC | GG | 552 |
| 15000509 | Non-gout | Male | 36 | TC | GG | 544 |
| 15000510 | Non-gout | Male | 59 | TC | AG | 539 |
| 15000511 | Non-gout | Male | 63 | TC | GG | 539 |
| 15000512 | Non-gout | Male | 64 | TC | GG | 537 |
| 15000513 | Non-gout | Female | 80 | TC | AG | 536 |
| 15000514 | Non-gout | Male | 69 | TC | GG | 529 |
| 15000515 | Non-gout | Male | 27 | TC | GG | 528 |
| 15000516 | Non-gout | Male | 74 | TC | AG | 527 |
| 15000517 | Non-gout | Female | 66 | TC | AA | 520 |
| 15000518 | Non-gout | Male | 66 | TC | AG | 518 |
| 15000519 | Non-gout | Female | 63 | TC | AG | 516 |
| 15000520 | Non-gout | Male | 40 | TC | GG | 515 |
| 15000521 | Non-gout | Male | 64 | TC | GG | 512 |
| 15000522 | Non-gout | Male | 67 | TC | AG | 509 |
| 15000523 | Non-gout | Male | 74 | TC | GG | 503 |
| 15000524 | Non-gout | Male | 59 | TC | AG | 503 |
| 15000525 | Non-gout | Female | 63 | TC | GG | 502 |
| 15000526 | Non-gout | Male | 29 | TC | AG | 502 |
| 15000527 | Non-gout | Male | 72 | TC | AA | 497 |
| 15000528 | Non-gout | Male | 74 | TC | GG | 492 |
| 15000529 | Non-gout | Female | 87 | TC | AG | 490 |
| 15000530 | Non-gout | Male | 85 | TC | GG | 490 |
| 15000531 | Non-gout | Male | 67 | TC | GG | 488 |
| 15000532 | Non-gout | Male | 66 | TC | GG | 488 |
| 15000533 | Non-gout | Male | 67 | TC | AG | 485 |
| 15000534 | Non-gout | Male | 80 | TC | AG | 485 |
| 15000535 | Non-gout | Male | 65 | TC | GG | 482 |
| 15000536 | Non-gout | Male | 80 | TC | GG | 481 |
| 15000537 | Non-gout | Male | 64 | TC | GG | 480 |
| 15000538 | Non-gout | Male | 80 | TC | AG | 480 |
| 15000539 | Non-gout | Male | 62 | TC | GG | 477 |
| 15000540 | Non-gout | Male | 71 | TC | AG | 476 |
| 15000541 | Non-gout | Male | 68 | TC | GG | 470 |
| 15000542 | Non-gout | Male | 28 | TC | GG | 469 |
| 15000543 | Non-gout | Female | 86 | TC | AG | 468 |
| 15000544 | Non-gout | Female | 83 | TC | GG | 467 |
| 15000545 | Non-gout | Male | 69 | TC | AG | 465 |
| 15000546 | Non-gout | Male | 70 | TC | GG | 464 |
| 15000547 | Non-gout | Male | 69 | TC | AG | 463 |
| 15000548 | Non-gout | Male | 57 | TC | GG | 461 |
| 15000549 | Non-gout | Female | 79 | TC | GG | 458 |
| 15000550 | Non-gout | Female | 85 | TC | GG | 457 |
| 15000551 | Non-gout | Female | 73 | TC | GG | 456 |
| 15000552 | Non-gout | Male | 49 | TC | AG | 456 |
| 15000553 | Non-gout | Male | 70 | TC | GG | 455 |
| 15000554 | Non-gout | Female | 83 | TC | AG | 454 |
| 15000555 | Non-gout | Male | 73 | TC | GG | 452 |
| 15000556 | Non-gout | Male | 79 | TC | AG | 450 |
| 15000557 | Non-gout | Female | 79 | TC | AG | 450 |
| 15000558 | Non-gout | Male | 41 | TC | AG | 449 |
| 15000559 | Non-gout | Male | 73 | TC | GG | 447 |
| 15000560 | Non-gout | Male | 67 | TC | AG | 447 |
| 15000561 | Non-gout | Female | 81 | TC | GG | 447 |
| 15000562 | Non-gout | Female | 80 | TC | GG | 446 |
| 15000563 | Non-gout | Male | 47 | TC | AG | 444 |
| 15000564 | Non-gout | Male | 68 | TC | GG | 442 |
| 15000565 | Non-gout | Male | 78 | TC | GG | 442 |
| 15000566 | Non-gout | Female | 63 | TC | GG | 442 |
| 15000567 | Non-gout | Male | 63 | TC | GG | 441 |
| 15000568 | Non-gout | Male | 69 | TC | GG | 440 |
| 15000569 | Non-gout | Male | 67 | TC | GG | 439 |
| 15000570 | Non-gout | Male | 83 | TC | AG | 438 |
| 15000571 | Non-gout | Male | 65 | TC | GG | 438 |
| 15000572 | Non-gout | Male | 65 | TC | AG | 438 |
| 15000573 | Non-gout | Male | 83 | TC | GG | 436 |
| 15000574 | Non-gout | Male | 64 | TC | GG | 436 |
| 15000575 | Non-gout | Female | 77 | TC | AG | 435 |
| 15000576 | Non-gout | Male | 68 | TC | GG | 434 |
| 15000577 | Non-gout | Male | 30 | TC | AA | 434 |
| 15000578 | Non-gout | Male | 75 | TC | AG | 433 |
| 15000579 | Non-gout | Female | 80 | TC | GG | 430 |
| 15000580 | Non-gout | Female | 82 | TC | AG | 430 |
| 15000581 | Non-gout | Female | 70 | TC | AG | 429 |
| 15000582 | Non-gout | Male | 80 | TC | GG | 428 |
| 15000583 | Non-gout | Male | 81 | TC | GG | 427 |
| 15000584 | Non-gout | Male | 35 | TC | GG | 427 |
| 15000585 | Non-gout | Male | 46 | TC | AG | 426 |
| 15000586 | Non-gout | Male | 70 | TC | GG | 425 |
| 15000587 | Non-gout | Male | 36 | TC | AG | 425 |
| 15000588 | Non-gout | Male | 73 | TC | AG | 424 |
| 15000589 | Non-gout | Male | 78 | TC | GG | 424 |
| 15000590 | Non-gout | Male | 76 | TC | AG | 424 |
| 15000591 | Non-gout | Male | 68 | TC | GG | 423 |
| 15000592 | Non-gout | Male | 67 | TC | AG | 422 |
| 15000593 | Non-gout | Male | 55 | TC | AG | 422 |
| 15000594 | Non-gout | Female | 81 | TC | AG | 421 |
| 15000595 | Non-gout | Male | 90 | TC | AG | 421 |
| 15000596 | Non-gout | Male | 72 | TC | AA | 420 |
| 15000597 | Non-gout | Female | 82 | TC | AG | 420 |
| 15000598 | Non-gout | Male | 53 | TC | AG | 415 |
| 15000599 | Non-gout | Male | 49 | TC | AG | 413 |
| 15000600 | Non-gout | Male | 49 | TC | AA | 412 |
| 15000601 | Non-gout | Male | 46 | TC | GG | 404 |
| 15000602 | Non-gout | Male |  | TC | GG | 401 |
| 15000603 | Non-gout | Female | 59 | TC | GG | 398 |
| 15000604 | Non-gout | Male | 32 | TC | GG | 397 |
| 15000605 | Non-gout | Male | 65 | TC | GG | 394 |
| 15000606 | Non-gout | Male | 29 | TC | GG | 389 |
| 15000607 | Non-gout | Male | 49 | TC | AG | 388 |
| 15000608 | Non-gout | Male | 26 | TC | AG | 388 |
| 15000609 | Non-gout | Male | 67 | TC | GG | 387 |
| 15000610 | Non-gout | Male | 26 | TC | AG | 380 |
| 15000611 | Non-gout | Male | 50 | TC | GG | 379 |
| 15000612 | Non-gout | Male |  | TC | GG | 378 |
| 15000613 | Non-gout | Male | 48 | TC | AG | 377 |
| 15000614 | Non-gout | Male | 56 | TC | AG | 376 |
| 15000615 | Non-gout | Male | 36 | TC | AA | 376 |
| 15000616 | Non-gout | Male | 59 | TC | GG | 374 |
| 15000617 | Non-gout | Female | 25 | TC | AG | 363 |
| 15000618 | Non-gout | Male | 35 | TC | AG | 360 |
| 15000619 | Non-gout | Male | 61 | TC | GG | 358 |
| 15000620 | Non-gout | Female | 24 | TC | GG | 356 |
| 15000621 | Non-gout | Male | 42 | TC | AG | 354 |
| 15000622 | Non-gout | Male | 74 | TC | GG | 353 |
| 15000623 | Non-gout | Male | 76 | TC | GG | 346 |
| 15000624 | Non-gout | Male | 57 | TC | GG | 346 |
| 15000625 | Non-gout | Male | 46 | TC | GG | 340 |
| 15000626 | Non-gout | Male | 59 | TC | GG | 336 |
| 15000627 | Non-gout | Male | 48 | TC | AG | 321 |
| 15000628 | Non-gout | Male | 29 | TC | AA | 321 |
| 15000629 | Non-gout | Male | 65 | TC | AG | 320 |
| 15000630 | Non-gout | Male | 41 | TC | AG | 319 |
| 15000631 | Non-gout | Male | 57 | TC | GG | 319 |
| 15000632 | Non-gout | Male | 83 | TC | AA | 319 |
| 15000633 | Non-gout | Male | 39 | TC | AG | 318 |
| 15000634 | Non-gout | Male | 29 | TC | AG | 311 |
| 15000635 | Non-gout | Male | 46 | TC | GG | 304 |
| 15000636 | Non-gout | Female | 64 | TC | GG | 288 |
| 15000637 | Non-gout | Male | 35 | TC | AG | 284 |
| 15000638 | Non-gout | Female | 24 | TC | AG | 277 |
| 15000639 | Non-gout | Male | 49 | TC | AG | 271 |
| 15000640 | Non-gout | Female | 40 | TC | AG | 261 |
| 15000641 | Non-gout | Female | 31 | TC | AA | 255 |
| 15000642 | Non-gout | Male | 60 | TC | GG | 250 |
| 15000643 | Non-gout | Female | 32 | TC | AG | 249 |
| 15000644 | Non-gout | Male | 52 | TC | GG | 235 |
| 15000645 | Non-gout |  |  | TC | AG | 233 |
| 15000646 | Non-gout | Female | 56 | TC | GG | 216 |
| 15000647 | Non-gout | Female | 24 | TC | AG | 214 |
| 15000648 | Non-gout | Female | 36 | TC | AG | 214 |
| 15000649 | Non-gout | Female | 41 | TC | GG | 209 |
| 15000650 | Non-gout | Female | 46 | TC | AG | 207 |
| 15000651 | Non-gout | Female | 29 | TC | AG | 204 |
| 15000652 | Non-gout | Female | 28 | TC | AG | 201 |
| 15000653 | Non-gout | Female | 38 | TC | GG | 198 |
| 15000654 | Non-gout | Female | 35 | TC | AG | 188 |
| 15000655 | Non-gout | Male | 34 | TC | GG | 186 |
| 15000656 | Non-gout | Female | 36 | TC | AG | 181 |
| 15000657 | Non-gout | Female | 39 | TC | GG | 177 |
| 15000658 | Non-gout | Female | 39 | TC | GG | 168 |
| 15000659 | Non-gout | Female | 36 | TC | AG | 146 |
| 15000660 | Non-gout | Male | 78 | TT | GG | 434 |
| 15000661 | Non-gout | Male | 73 | TT | AG | 398 |
| 15000662 | Non-gout | Male | 69 | TT | GG | 372 |
| 15000663 | Non-gout | Male | 66 | TT | AA | 548 |
| 15000664 | Non-gout | Male | 75 | TT | AA | 396 |
| 15000665 | Non-gout | Male | 73 | TT | GG | 326 |
| 15000666 | Non-gout | Male | 66 | TT | GG | 463 |
| 15000667 | Non-gout | Female | 90 | TT | GG | 425 |
| 15000668 | Non-gout | Male | 74 | TT | AA | 521 |
| 15000669 | Non-gout | Male | 64 | TT | GG | 431 |
| 15000670 | Non-gout | Male | 61 | TT | AG | 360 |
| 15000671 | Non-gout | Male | 64 | TT | AA | 429 |
| 15000672 | Non-gout | Male | 70 | TT | AG | 590 |
| 15000673 | Non-gout | Male | 59 | TT | AA | 531 |
| 15000674 | Non-gout | Male | 68 | TT | AG | 320 |
| 15000675 | Non-gout | Male | 64 | TT | GG | 439 |
| 15000676 | Non-gout | Male | 71 | TT | GG | 612 |
| 15000677 | Non-gout | Female | 80 | TT | AG | 455 |
| 15000678 | Non-gout | Male | 65 | TT | AG | 427 |
| 15000679 | Non-gout | Female | 60 | TT | GG | 540 |
| 15000680 | Non-gout | Female | 67 | TT | AG | 464 |
| 15000681 | Non-gout | Female | 62 | TT | AG | 461 |
| 15000682 | Non-gout | Male | 70 | TT | AG | 269 |
| 15000683 | Non-gout | Female | 71 | TT | GG | 424 |
| 15000684 | Non-gout | Male | 63 | TT | GG | 232 |
| 15000685 | Non-gout | Male | 77 | TT | GG | 323 |
| 15000686 | Non-gout | Female | 57 | TT | AG | 470 |
| 15000687 | Non-gout | Male | 66 | TT | AG | 593 |
| 15000688 | Non-gout | Male | 69 | TT | GG | 452 |
| 15000689 | Non-gout | Male | 67 | TT | GG | 302 |
| 15000690 | Non-gout | Male | 61 | TT | GG | 335 |
| 15000691 | Non-gout | Female | 64 | TT | GG | 305 |
| 15000692 | Non-gout | Male | 66 | TT | GG | 292 |
| 15000693 | Non-gout | Male | 66 | TT | GG | 369 |
| 15000694 | Non-gout | Female | 62 | TT | AG | 200 |
| 15000695 | Non-gout | Male | 74 | TT | AA | 299 |
| 15000696 | Non-gout | Male | 75 | TT | GG | 314 |
| 15000697 | Non-gout | Male | 71 | TT | GG | 537 |
| 15000698 | Non-gout | Male | 71 | TT | AA | 441 |
| 15000699 | Non-gout | Male | 69 | TT | GG | 488 |
| 15000700 | Non-gout | Male | 71 | TT | AG | 486 |
| 15000701 | Non-gout | Male | 83 | TT | AA | 471 |
| 15000702 | Non-gout | Female | 56 | TT | AG | 485 |
| 15000703 | Non-gout | Male | 69 | TT | AG | 436 |
| 15000704 | Non-gout | Female | 58 | TT | GG | 232 |
| 15000705 | Non-gout | Male | 71 | TT | GG | 360 |
| 15000706 | Non-gout | Male | 72 | TT | GG | 657 |
| 15000707 | Non-gout | Male | 64 | TT | AG | 420 |
| 15000708 | Non-gout | Female | 66 | TT | GG | 355 |
| 15000709 | Non-gout | Female | 59 | TT | AA | 221 |
| 15000710 | Non-gout | Male | 78 | TT | AG | 450 |
| 15000711 | Non-gout | Female | 58 | TT | GG | 442 |
| 15000712 | Non-gout | Male | 68 | TT | AG | 262 |
| 15000713 | Non-gout | Male | 70 | TT | AG | 440 |
| 15000714 | Non-gout | Male | 103 | TT | AG | 434 |
| 15000715 | Non-gout | Female | 70 | TT | GG | 278 |
| 15000716 | Non-gout | Male | 77 | TT | GG | 492 |
| 15000717 | Non-gout | Male | 79 | TT | GG | 473 |
| 15000718 | Non-gout | Male | 60 | TT | AG | 424 |
| 15000719 | Non-gout | Female | 61 | TT | AG | 349 |
| 15000720 | Non-gout | Female | 50 | TT | GG | 529 |
| 15000721 | Non-gout | Male | 58 | TT | AG | 451 |
| 15000722 | Non-gout | Male | 77 | TT | GG | 227 |
| 15000723 | Non-gout | Male | 67 | TT | AG | 323 |
| 15000724 | Non-gout | Female | 64 | TT | GG | 335 |
| 15000725 | Non-gout | Male | 69 | TT | GG | 537 |
| 15000726 | Non-gout | Male | 65 | TT | GG | 518 |
| 15000727 | Non-gout | Male | 93 | TT | GG | 471 |
| 15000728 | Non-gout | Female | 60 | TT | AG | 254 |
| 15000729 | Non-gout | Female | 67 | TT | AG | 246 |
| 15000730 | Non-gout | Male | 67 | TT | GG | 443 |
| 15000731 | Non-gout | Male | 83 | TT | AG | 336 |
| 15000732 | Non-gout | Male | 70 | TT | GG | 346 |
| 15000733 | Non-gout | Female | 68 | TT | GG | 268 |
| 15000734 | Non-gout | Female | 65 | TT | AG | 253 |
| 15000735 | Non-gout | Male | 63 | TT | AG | 552 |
| 15000736 | Non-gout | Male | 64 | TT | GG | 507 |
| 15000737 | Non-gout | Male | 82 | TT | AA | 234 |
| 15000738 | Non-gout | Female | 73 | TT | AG | 450 |
| 15000739 | Non-gout | Male | 69 | TT | GG | 273 |
| 15000740 | Non-gout | Male | 74 | TT | AG | 275 |
| 15000741 | Non-gout | Male | 61 | TT | GG | 332 |
| 15000742 | Non-gout | Male | 65 | TT | GG | 321 |
| 15000743 | Non-gout | Female | 78 | TT | AG | 490 |
| 15000744 | Non-gout | Male | 65 | TT | AG | 395 |
| 15000745 | Non-gout | Male | 71 | TT | GG | 232 |
| 15000746 | Non-gout | Male | 72 | TT | GG | 356 |
| 15000747 | Non-gout | Male | 61 | TT | GG | 273 |
| 15000748 | Non-gout | Male | 68 | TT | AG | 172 |
| 15000749 | Non-gout | Male | 67 | TT | GG | 296 |
| 15000750 | Non-gout | Female | 59 | TT | AG | 269 |
| 15000751 | Non-gout | Male | 60 | TT | GG | 442 |
| 15000752 | Non-gout | Female | 64 | TT | AG | 462 |
| 15000753 | Non-gout | Male | 77 | TT | AG | 508 |
| 15000754 | Non-gout | Male | 68 | TT | AG | 250 |
| 15000755 | Non-gout | Male | 75 | TT | GG | 221 |
| 15000756 | Non-gout | Female | 72 | TT | AG | 437 |
| 15000757 | Non-gout | Male | 66 | TT | AG | 650 |
| 15000758 | Non-gout | Female | 63 | TT | AG | 592 |
| 15000759 | Non-gout | Male | 74 | TT | GG | 581 |
| 15000760 | Non-gout | Male | 67 | TT | GG | 557 |
| 15000761 | Non-gout | Male | 66 | TT | GG | 537 |
| 15000762 | Non-gout | Male | 69 | TT | AG | 515 |
| 15000763 | Non-gout | Male | 84 | TT | AG | 514 |
| 15000764 | Non-gout | Male | 67 | TT | GG | 511 |
| 15000765 | Non-gout | Female | 79 | TT | AG | 508 |
| 15000766 | Non-gout | Male | 83 | TT | GG | 505 |
| 15000767 | Non-gout | Male | 72 | TT | GG | 499 |
| 15000768 | Non-gout | Female | 86 | TT | AG | 494 |
| 15000769 | Non-gout | Male | 62 | TT | GG | 492 |
| 15000770 | Non-gout | Male | 64 | TT | AG | 491 |
| 15000771 | Non-gout | Female | 79 | TT | AG | 487 |
| 15000772 | Non-gout | Male | 73 | TT | GG | 486 |
| 15000773 | Non-gout | Female | 63 | TT | AG | 486 |
| 15000774 | Non-gout | Male | 47 | TT | GG | 483 |
| 15000775 | Non-gout | Male | 67 | TT | GG | 476 |
| 15000776 | Non-gout | Male | 38 | TT | GG | 475 |
| 15000777 | Non-gout | Male | 68 | TT | GG | 472 |
| 15000778 | Non-gout | Male | 81 | TT | AG | 471 |
| 15000779 | Non-gout | Male | 52 | TT | AG | 466 |
| 15000780 | Non-gout | Male | 63 | TT | AG | 465 |
| 15000781 | Non-gout | Male | 71 | TT | GG | 464 |
| 15000782 | Non-gout | Male | 82 | TT | GG | 461 |
| 15000783 | Non-gout | Male | 65 | TT | AG | 461 |
| 15000784 | Non-gout | Male | 83 | TT | GG | 460 |
| 15000785 | Non-gout | Male | 62 | TT | AA | 459 |
| 15000786 | Non-gout | Male | 49 | TT | AG | 459 |
| 15000787 | Non-gout | Male | 89 | TT | GG | 456 |
| 15000788 | Non-gout | Male | 58 | TT | AA | 455 |
| 15000789 | Non-gout | Male | 83 | TT | AG | 453 |
| 15000790 | Non-gout | Male | 59 | TT | GG | 453 |
| 15000791 | Non-gout | Male | 63 | TT | GG | 452 |
| 15000792 | Non-gout | Male | 77 | TT | AA | 449 |
| 15000793 | Non-gout | Male | 62 | TT | GG | 447 |
| 15000794 | Non-gout | Male | 64 | TT | GG | 445 |
| 15000795 | Non-gout | Male | 71 | TT | AG | 445 |
| 15000796 | Non-gout | Male | 50 | TT | AG | 444 |
| 15000797 | Non-gout | Male | 78 | TT | AG | 441 |
| 15000798 | Non-gout | Male | 66 | TT | GG | 441 |
| 15000799 | Non-gout | Male | 72 | TT | AA | 435 |
| 15000800 | Non-gout | Male | 51 | TT | AG | 435 |
| 15000801 | Non-gout | Male | 57 | TT | GG | 434 |
| 15000802 | Non-gout | Female | 82 | TT | AG | 432 |
| 15000803 | Non-gout | Male | 73 | TT | AG | 431 |
| 15000804 | Non-gout | Male | 63 | TT | AG | 431 |
| 15000805 | Non-gout | Female | 74 | TT | GG | 429 |
| 15000806 | Non-gout | Male | 63 | TT | AG | 427 |
| 15000807 | Non-gout | Male | 84 | TT | GG | 426 |
| 15000808 | Non-gout | Male | 49 | TT | AG | 425 |
| 15000809 | Non-gout | Male | 31 | TT | GG | 425 |
| 15000810 | Non-gout | Male | 50 | TT | AG | 425 |
| 15000811 | Non-gout | Male | 69 | TT | AG | 423 |
| 15000812 | Non-gout | Female | 83 | TT | GG | 423 |
| 15000813 | Non-gout | Male | 84 | TT | GG | 420 |
| 15000814 | Non-gout | Male | 50 | TT | GG | 412 |
| 15000815 | Non-gout | Male | 41 | TT | AG | 396 |
| 15000816 | Non-gout | Male | 37 | TT | GG | 394 |
| 15000817 | Non-gout | Male | 43 | TT | GG | 391 |
| 15000818 | Non-gout | Female | 74 | TT | GG | 387 |
| 15000819 | Non-gout | Male | 66 | TT | AG | 385 |
| 15000820 | Non-gout | Male | 62 | TT | AA | 380 |
| 15000821 | Non-gout | Female | 54 | TT | AG | 373 |
| 15000822 | Non-gout | Male | 36 | TT | GG | 371 |
| 15000823 | Non-gout | Male | 39 | TT | AG | 368 |
| 15000824 | Non-gout | Male |  | TT | AG | 342 |
| 15000825 | Non-gout | Male | 59 | TT | GG | 276 |
| 15000826 | Non-gout | Male | 44 | TT | GG | 273 |
| 15000827 | Non-gout | Male | 45 | TT | GG | 267 |
| 15000828 | Non-gout | Female | 41 | TT | GG | 251 |
| 15000829 | Non-gout | Female | 36 | TT | AG | 228 |
| 15000830 | Non-gout | Male | 39 | TT | AG | 227 |
| 15000831 | Non-gout | Female | 49 | TT | AG | 214 |
| 15000832 | Non-gout | Female | 27 | TT | GG | 201 |
| 15000833 | Non-gout | Male | 43 | TT | AG | 199 |
| 15000834 | Non-gout | Female | 47 | TT | GG | 178 |
| 15000835 | Non-gout | Female | 44 | TT | AG | 178 |
| 15000836 | Gout |  |  | CC | GG |  |
| 15000837 | Gout |  |  | CC | AG |  |
| 15000838 | Gout |  |  | CC | GG |  |
| 15000839 | Gout |  |  | CC | GG |  |
| 15000840 | Gout |  |  | CC | GG |  |
| 15000841 | Gout |  |  | CC | GG |  |
| 15000842 | Gout |  |  | CC | GG |  |
| 15000843 | Gout |  |  | CC | GG |  |
| 15000844 | Gout |  |  | CC | GG |  |
| 15000845 | Gout |  |  | CC | GG |  |
| 15000846 | Gout |  |  | CC | AG |  |
| 15000847 | Gout |  |  | CC | GG |  |
| 15000848 | Gout |  |  | CC | AG |  |
| 15000849 | Gout |  |  | CC | GG |  |
| 15000850 | Gout |  |  | CC | AG |  |
| 15000851 | Gout |  |  | CC | AG |  |
| 15000852 | Gout |  |  | CC | AG |  |
| 15000853 | Gout |  |  | CC | AG |  |
| 15000854 | Gout |  |  | CC | AA |  |
| 15000855 | Gout |  |  | CC | AG |  |
| 15000856 | Gout |  |  | CC | GG |  |
| 15000857 | Gout |  |  | CC | AA |  |
| 15000858 | Gout |  |  | CC | GG |  |
| 15000859 | Gout |  |  | CC | GG |  |
| 15000860 | Gout |  |  | CC | GG |  |
| 15000861 | Gout |  |  | CC | GG |  |
| 15000862 | Gout |  |  | CC | GG |  |
| 15000863 | Gout |  |  | CC | AA |  |
| 15000864 | Gout |  |  | CC | GG |  |
| 15000865 | Gout |  |  | CC | GG |  |
| 15000866 | Gout |  |  | CC | AG |  |
| 15000867 | Gout |  |  | CC | AA |  |
| 15000868 | Gout |  |  | CC | GG |  |
| 15000869 | Gout |  |  | CC | GG |  |
| 15000870 | Gout |  |  | CC | GG |  |
| 15000871 | Gout |  |  | CC | GG |  |
| 15000872 | Gout |  |  | CC | GG |  |
| 15000873 | Gout |  |  | CC | AG |  |
| 15000874 | Gout |  |  | CC | AG |  |
| 15000875 | Gout |  |  | CC | GG |  |
| 15000876 | Gout |  |  | CC | GG |  |
| 15000877 | Gout |  |  | CC | GG |  |
| 15000878 | Gout |  |  | CC | AA |  |
| 15000879 | Gout |  |  | CC | GG |  |
| 15000880 | Gout |  |  | CC | GG |  |
| 15000881 | Gout |  |  | CC | AG |  |
| 15000882 | Gout |  |  | CC | GG |  |
| 15000883 | Gout |  |  | CC | GG |  |
| 15000884 | Gout |  |  | CC | AG |  |
| 15000885 | Gout |  |  | CC | GG |  |
| 15000886 | Gout |  |  | CC | GG |  |
| 15000887 | Gout |  |  | CC | GG |  |
| 15000888 | Gout |  |  | CC | GG |  |
| 15000889 | Gout |  |  | CC | AG |  |
| 15000890 | Gout |  |  | CC | GG |  |
| 15000891 | Gout |  |  | CC | AG |  |
| 15000892 | Gout |  |  | CC | AG |  |
| 15000893 | Gout |  |  | CC | GG |  |
| 15000894 | Gout |  |  | CC | AG |  |
| 15000895 | Gout |  |  | CC | AG |  |
| 15000896 | Gout |  |  | CC | GG |  |
| 15000897 | Gout |  |  | CC | AG |  |
| 15000898 | Gout |  |  | CC | GG |  |
| 15000899 | Gout |  |  | CC | AG |  |
| 15000900 | Gout |  |  | CC | GG |  |
| 15000901 | Gout |  |  | CC | AA |  |
| 15000902 | Gout |  |  | CC | AG |  |
| 15000903 | Gout |  |  | CC | AG |  |
| 15000904 | Gout |  |  | CC | AA |  |
| 15000905 | Gout |  |  | CC | AA |  |
| 15000906 | Gout |  |  | CC | AG |  |
| 15000907 | Gout |  |  | CC | AG |  |
| 15000908 | Gout |  |  | CC | GG |  |
| 15000909 | Gout |  |  | CC | GG |  |
| 15000910 | Gout |  |  | CC | AG |  |
| 15000911 | Gout |  |  | CC | GG |  |
| 15000912 | Gout |  |  | CC | GG |  |
| 15000913 | Gout |  |  | CC | AG |  |
| 15000914 | Gout |  |  | CC | AG |  |
| 15000915 | Gout |  |  | CC | GG |  |
| 15000916 | Gout |  |  | CC | GG |  |
| 15000917 | Gout |  |  | CC | AA |  |
| 15000918 | Gout |  |  | CC | GG |  |
| 15000919 | Gout |  |  | CC | AG |  |
| 15000920 | Gout |  |  | CC | GG |  |
| 15000921 | Gout |  |  | CC | GG |  |
| 15000922 | Gout |  |  | CC | GG |  |
| 15000923 | Gout |  |  | CC | GG |  |
| 15000924 | Gout |  |  | CC | GG |  |
| 15000925 | Gout |  |  | CC | AG |  |
| 15000926 | Gout |  |  | CC | AG |  |
| 15000927 | Gout |  |  | CC | GG |  |
| 15000928 | Gout |  |  | CC | AG |  |
| 15000929 | Gout |  |  | CC | GG |  |
| 15000930 | Gout |  |  | CC | GG |  |
| 15000931 | Gout |  |  | CC | AG |  |
| 15000932 | Gout |  |  | CC | AG |  |
| 15000933 | Gout |  |  | CC | GG |  |
| 15000934 | Gout |  |  | CC | GG |  |
| 15000935 | Gout |  |  | CC | AG |  |
| 15000936 | Gout |  |  | CC | AG |  |
| 15000937 | Gout |  |  | CC | AG |  |
| 15000938 | Gout |  |  | CC | GG |  |
| 15000939 | Gout |  |  | CC | AG |  |
| 15000940 | Gout |  |  | CC | GG |  |
| 15000941 | Gout |  |  | CC | GG |  |
| 15000942 | Gout |  |  | CC | GG |  |
| 15000943 | Gout |  |  | CC | GG |  |
| 15000944 | Gout |  |  | CC | AG |  |
| 15000945 | Gout |  |  | CC | GG |  |
| 15000946 | Gout |  |  | CC | AG |  |
| 15000947 | Gout |  |  | CC | GG |  |
| 15000948 | Gout |  |  | CC | GG |  |
| 15000949 | Gout |  |  | CC | AG |  |
| 15000950 | Gout |  |  | CC | AG |  |
| 15000951 | Gout |  |  | CC | GG |  |
| 15000952 | Gout |  |  | CC | AG |  |
| 15000953 | Gout |  |  | CC | GG |  |
| 15000954 | Gout |  |  | CC | AG |  |
| 15000955 | Gout |  |  | CC | AG |  |
| 15000956 | Gout |  |  | CC | AG |  |
| 15000957 | Gout |  |  | CC | AG |  |
| 15000958 | Gout |  |  | CC | AG |  |
| 15000959 | Gout |  |  | CC | GG |  |
| 15000960 | Gout |  |  | CC | GG |  |
| 15000961 | Gout |  |  | CC | GG |  |
| 15000962 | Gout |  |  | CC | AG |  |
| 15000963 | Gout |  |  | CC | GG |  |
| 15000964 | Gout |  |  | CC | GG |  |
| 15000965 | Gout |  |  | CC | AG |  |
| 15000966 | Gout |  |  | CC | AG |  |
| 15000967 | Gout |  |  | CC | GG |  |
| 15000968 | Gout |  |  | CC | GG |  |
| 15000969 | Gout |  |  | TC | GG |  |
| 15000970 | Gout |  |  | TC | GG |  |
| 15000971 | Gout |  |  | TC | AG |  |
| 15000972 | Gout |  |  | TC | GG |  |
| 15000973 | Gout |  |  | TC | GG |  |
| 15000974 | Gout |  |  | TC | AG |  |
| 15000975 | Gout |  |  | TC | AA |  |
| 15000976 | Gout |  |  | TC | AA |  |
| 15000977 | Gout |  |  | TC | GG |  |
| 15000978 | Gout |  |  | TC | GG |  |
| 15000979 | Gout |  |  | TC | GG |  |
| 15000980 | Gout |  |  | TC | GG |  |
| 15000981 | Gout |  |  | TC | GG |  |
| 15000982 | Gout |  |  | TC | GG |  |
| 15000983 | Gout |  |  | TC | GG |  |
| 15000984 | Gout |  |  | TC | GG |  |
| 15000985 | Gout |  |  | TC | AG |  |
| 15000986 | Gout |  |  | TC | AG |  |
| 15000987 | Gout |  |  | TC | AG |  |
| 15000988 | Gout |  |  | TC | GG |  |
| 15000989 | Gout |  |  | TC | GG |  |
| 15000990 | Gout |  |  | TC | GG |  |
| 15000991 | Gout |  |  | TC | AG |  |
| 15000992 | Gout |  |  | TC | AG |  |
| 15000993 | Gout |  |  | TC | AG |  |
| 15000994 | Gout |  |  | TC | AG |  |
| 15000995 | Gout |  |  | TC | AG |  |
| 15000996 | Gout |  |  | TC | AG |  |
| 15000997 | Gout |  |  | TC | AG |  |
| 15000998 | Gout |  |  | TC | GG |  |
| 15000999 | Gout |  |  | TC | GG |  |
| 15001000 | Gout |  |  | TC | AG |  |
| 15001001 | Gout |  |  | TC | AG |  |
| 15001002 | Gout |  |  | TC | GG |  |
| 15001003 | Gout |  |  | TC | GG |  |
| 15001004 | Gout |  |  | TC | AG |  |
| 15001005 | Gout |  |  | TC | AA |  |
| 15001006 | Gout |  |  | TC | GG |  |
| 15001007 | Gout |  |  | TC | GG |  |
| 15001008 | Gout |  |  | TC | AG |  |
| 15001009 | Gout |  |  | TC | AG |  |
| 15001010 | Gout |  |  | TC | AG |  |
| 15001011 | Gout |  |  | TC | AG |  |
| 15001012 | Gout |  |  | TC | GG |  |
| 15001013 | Gout |  |  | TC | GG |  |
| 15001014 | Gout |  |  | TC | GG |  |
| 15001015 | Gout |  |  | TC | AA |  |
| 15001016 | Gout |  |  | TC | GG |  |
| 15001017 | Gout |  |  | TC | AG |  |
| 15001018 | Gout |  |  | TC | AA |  |
| 15001019 | Gout |  |  | TC | GG |  |
| 15001020 | Gout |  |  | TC | AG |  |
| 15001021 | Gout |  |  | TC | GG |  |
| 15001022 | Gout |  |  | TC | AG |  |
| 15001023 | Gout |  |  | TC | GG |  |
| 15001024 | Gout |  |  | TC | AG |  |
| 15001025 | Gout |  |  | TC | GG |  |
| 15001026 | Gout |  |  | TC | AG |  |
| 15001027 | Gout |  |  | TC | GG |  |
| 15001028 | Gout |  |  | TC | GG |  |
| 15001029 | Gout |  |  | TC | AG |  |
| 15001030 | Gout |  |  | TC | GG |  |
| 15001031 | Gout |  |  | TC | AG |  |
| 15001032 | Gout |  |  | TC | GG |  |
| 15001033 | Gout |  |  | TC | GG |  |
| 15001034 | Gout |  |  | TC | GG |  |
| 15001035 | Gout |  |  | TC | AG |  |
| 15001036 | Gout |  |  | TC | GG |  |
| 15001037 | Gout |  |  | TC | GG |  |
| 15001038 | Gout |  |  | TC | GG |  |
| 15001039 | Gout |  |  | TC | GG |  |
| 15001040 | Gout |  |  | TC | GG |  |
| 15001041 | Gout |  |  | TC | AG |  |
| 15001042 | Gout |  |  | TC | GG |  |
| 15001043 | Gout |  |  | TC | AG |  |
| 15001044 | Gout |  |  | TC | AG |  |
| 15001045 | Gout |  |  | TC | AG |  |
| 15001046 | Gout |  |  | TC | GG |  |
| 15001047 | Gout |  |  | TC | GG |  |
| 15001048 | Gout |  |  | TC | AG |  |
| 15001049 | Gout |  |  | TC | AG |  |
| 15001050 | Gout |  |  | TC | AA |  |
| 15001051 | Gout |  |  | TC | GG |  |
| 15001052 | Gout |  |  | TC | GG |  |
| 15001053 | Gout |  |  | TC | GG |  |
| 15001054 | Gout |  |  | TC | GG |  |
| 15001055 | Gout |  |  | TC | GG |  |
| 15001056 | Gout |  |  | TC | AG |  |
| 15001057 | Gout |  |  | TC | AA |  |
| 15001058 | Gout |  |  | TC | AG |  |
| 15001059 | Gout |  |  | TC | GG |  |
| 15001060 | Gout |  |  | TC | AG |  |
| 15001061 | Gout |  |  | TC | GG |  |
| 15001062 | Gout |  |  | TC | AG |  |
| 15001063 | Gout |  |  | TC | GG |  |
| 15001064 | Gout |  |  | TC | GG |  |
| 15001065 | Gout |  |  | TC | AG |  |
| 15001066 | Gout |  |  | TC | AA |  |
| 15001067 | Gout |  |  | TC | AG |  |
| 15001068 | Gout |  |  | TC | AA |  |
| 15001069 | Gout |  |  | TC | AG |  |
| 15001070 | Gout |  |  | TC | GG |  |
| 15001071 | Gout |  |  | TC | AG |  |
| 15001072 | Gout |  |  | TC | GG |  |
| 15001073 | Gout |  |  | TC | AG |  |
| 15001074 | Gout |  |  | TC | GG |  |
| 15001075 | Gout |  |  | TC | AG |  |
| 15001076 | Gout |  |  | TC | GG |  |
| 15001077 | Gout |  |  | TC | GG |  |
| 15001078 | Gout |  |  | TC | AG |  |
| 15001079 | Gout |  |  | TC | AG |  |
| 15001080 | Gout |  |  | TC | AG |  |
| 15001081 | Gout |  |  | TC | GG |  |
| 15001082 | Gout |  |  | TC | AG |  |
| 15001083 | Gout |  |  | TC | GG |  |
| 15001084 | Gout |  |  | TC | GG |  |
| 15001085 | Gout |  |  | TC | GG |  |
| 15001086 | Gout |  |  | TC | GG |  |
| 15001087 | Gout |  |  | TC | GG |  |
| 15001088 | Gout |  |  | TC | GG |  |
| 15001089 | Gout |  |  | TC | GG |  |
| 15001090 | Gout |  |  | TC | GG |  |
| 15001091 | Gout |  |  | TC | AG |  |
| 15001092 | Gout |  |  | TC | GG |  |
| 15001093 | Gout |  |  | TC | AG |  |
| 15001094 | Gout |  |  | TC | GG |  |
| 15001095 | Gout |  |  | TC | GG |  |
| 15001096 | Gout |  |  | TC | GG |  |
| 15001097 | Gout |  |  | TC | GG |  |
| 15001098 | Gout |  |  | TC | GG |  |
| 15001099 | Gout |  |  | TC | GG |  |
| 15001100 | Gout |  |  | TC | GG |  |
| 15001101 | Gout |  |  | TC | AG |  |
| 15001102 | Gout |  |  | TC | GG |  |
| 15001103 | Gout |  |  | TC | AG |  |
| 15001104 | Gout |  |  | TC | AG |  |
| 15001105 | Gout |  |  | TC | GG |  |
| 15001106 | Gout |  |  | TC | AA |  |
| 15001107 | Gout |  |  | TC | AG |  |
| 15001108 | Gout |  |  | TC | GG |  |
| 15001109 | Gout |  |  | TC | AG |  |
| 15001110 | Gout |  |  | TC | GG |  |
| 15001111 | Gout |  |  | TC | GG |  |
| 15001112 | Gout |  |  | TC | AG |  |
| 15001113 | Gout |  |  | TC | AG |  |
| 15001114 | Gout |  |  | TC | GG |  |
| 15001115 | Gout |  |  | TC | AA |  |
| 15001116 | Gout |  |  | TC | GG |  |
| 15001117 | Gout |  |  | TC | GG |  |
| 15001118 | Gout |  |  | TC | AA |  |
| 15001119 | Gout |  |  | TC | GG |  |
| 15001120 | Gout |  |  | TC | AG |  |
| 15001121 | Gout |  |  | TC | GG |  |
| 15001122 | Gout |  |  | TC | GG |  |
| 15001123 | Gout |  |  | TC | GG |  |
| 15001124 | Gout |  |  | TC | GG |  |
| 15001125 | Gout |  |  | TC | GG |  |
| 15001126 | Gout |  |  | TC | GG |  |
| 15001127 | Gout |  |  | TC | AG |  |
| 15001128 | Gout |  |  | TC | AG |  |
| 15001129 | Gout |  |  | TC | GG |  |
| 15001130 | Gout |  |  | TC | AG |  |
| 15001131 | Gout |  |  | TC | AG |  |
| 15001132 | Gout |  |  | TC | AG |  |
| 15001133 | Gout |  |  | TC | AG |  |
| 15001134 | Gout |  |  | TC | GG |  |
| 15001135 | Gout |  |  | TC | AG |  |
| 15001136 | Gout |  |  | TC | AG |  |
| 15001137 | Gout |  |  | TC | AG |  |
| 15001138 | Gout |  |  | TC | AA |  |
| 15001139 | Gout |  |  | TC | AG |  |
| 15001140 | Gout |  |  | TC | AA |  |
| 15001141 | Gout |  |  | TC | AA |  |
| 15001142 | Gout |  |  | TC | AG |  |
| 15001143 | Gout |  |  | TC | GG |  |
| 15001144 | Gout |  |  | TC | AG |  |
| 15001145 | Gout |  |  | TC | GG |  |
| 15001146 | Gout |  |  | TC | AG |  |
| 15001147 | Gout |  |  | TC | AG |  |
| 15001148 | Gout |  |  | TC | GG |  |
| 15001149 | Gout |  |  | TC | GG |  |
| 15001150 | Gout |  |  | TC | AG |  |
| 15001151 | Gout |  |  | TC | AG |  |
| 15001152 | Gout |  |  | TC | GG |  |
| 15001153 | Gout |  |  | TC | GG |  |
| 15001154 | Gout |  |  | TC | AG |  |
| 15001155 | Gout |  |  | TC | AA |  |
| 15001156 | Gout |  |  | TC | AG |  |
| 15001157 | Gout |  |  | TC | AG |  |
| 15001158 | Gout |  |  | TC | AG |  |
| 15001159 | Gout |  |  | TC | GG |  |
| 15001160 | Gout |  |  | TC | GG |  |
| 15001161 | Gout |  |  | TC | GG |  |
| 15001162 | Gout |  |  | TC | GG |  |
| 15001163 | Gout |  |  | TC | AG |  |
| 15001164 | Gout |  |  | TC | GG |  |
| 15001165 | Gout |  |  | TC | AG |  |
| 15001166 | Gout |  |  | TC | GG |  |
| 15001167 | Gout |  |  | TC | GG |  |
| 15001168 | Gout |  |  | TC | GG |  |
| 15001169 | Gout |  |  | TC | GG |  |
| 15001170 | Gout |  |  | TC | GG |  |
| 15001171 | Gout |  |  | TC | AG |  |
| 15001172 | Gout |  |  | TC | AG |  |
| 15001173 | Gout |  |  | TC | GG |  |
| 15001174 | Gout |  |  | TC | AG |  |
| 15001175 | Gout |  |  | TC | GG |  |
| 15001176 | Gout |  |  | TC | GG |  |
| 15001177 | Gout |  |  | TC | GG |  |
| 15001178 | Gout |  |  | TC | GG |  |
| 15001179 | Gout |  |  | TC | AG |  |
| 15001180 | Gout |  |  | TC | AG |  |
| 15001181 | Gout |  |  | TC | AG |  |
| 15001182 | Gout |  |  | TC | GG |  |
| 15001183 | Gout |  |  | TC | AG |  |
| 15001184 | Gout |  |  | TC | GG |  |
| 15001185 | Gout |  |  | TC | GG |  |
| 15001186 | Gout |  |  | TC | AG |  |
| 15001187 | Gout |  |  | TC | GG |  |
| 15001188 | Gout |  |  | TC | GG |  |
| 15001189 | Gout |  |  | TC | AG |  |
| 15001190 | Gout |  |  | TC | AG |  |
| 15001191 | Gout |  |  | TC | GG |  |
| 15001192 | Gout |  |  | TC | AG |  |
| 15001193 | Gout |  |  | TC | GG |  |
| 15001194 | Gout |  |  | TC | GG |  |
| 15001195 | Gout |  |  | TC | GG |  |
| 15001196 | Gout |  |  | TC | AG |  |
| 15001197 | Gout |  |  | TC | AG |  |
| 15001198 | Gout |  |  | TC | AG |  |
| 15001199 | Gout |  |  | TC | GG |  |
| 15001200 | Gout |  |  | TC | GG |  |
| 15001201 | Gout |  |  | TC | AA |  |
| 15001202 | Gout |  |  | TC | AG |  |
| 15001203 | Gout |  |  | TC | AG |  |
| 15001204 | Gout |  |  | TC | AG |  |
| 15001205 | Gout |  |  | TC | GG |  |
| 15001206 | Gout |  |  | TC | GG |  |
| 15001207 | Gout |  |  | TC | AG |  |
| 15001208 | Gout |  |  | TC | AG |  |
| 15001209 | Gout |  |  | TC | AG |  |
| 15001210 | Gout |  |  | TT | GG |  |
| 15001211 | Gout |  |  | TT | GG |  |
| 15001212 | Gout |  |  | TT | GG |  |
| 15001213 | Gout |  |  | TT | GG |  |
| 15001214 | Gout |  |  | TT | AG |  |
| 15001215 | Gout |  |  | TT | AG |  |
| 15001216 | Gout |  |  | TT | GG |  |
| 15001217 | Gout |  |  | TT | GG |  |
| 15001218 | Gout |  |  | TT | AG |  |
| 15001219 | Gout |  |  | TT | AG |  |
| 15001220 | Gout |  |  | TT | GG |  |
| 15001221 | Gout |  |  | TT | GG |  |
| 15001222 | Gout |  |  | TT | AG |  |
| 15001223 | Gout |  |  | TT | AG |  |
| 15001224 | Gout |  |  | TT | GG |  |
| 15001225 | Gout |  |  | TT | GG |  |
| 15001226 | Gout |  |  | TT | GG |  |
| 15001227 | Gout |  |  | TT | GG |  |
| 15001228 | Gout |  |  | TT | GG |  |
| 15001229 | Gout |  |  | TT | AG |  |
| 15001230 | Gout |  |  | TT | GG |  |
| 15001231 | Gout |  |  | TT | GG |  |
| 15001232 | Gout |  |  | TT | GG |  |
| 15001233 | Gout |  |  | TT | AG |  |
| 15001234 | Gout |  |  | TT | GG |  |
| 15001235 | Gout |  |  | TT | AG |  |
| 15001236 | Gout |  |  | TT | AG |  |
| 15001237 | Gout |  |  | TT | GG |  |
| 15001238 | Gout |  |  | TT | GG |  |
| 15001239 | Gout |  |  | TT | GG |  |
| 15001240 | Gout |  |  | TT | GG |  |
| 15001241 | Gout |  |  | TT | GG |  |
| 15001242 | Gout |  |  | TT | GG |  |
| 15001243 | Gout |  |  | TT | GG |  |
| 15001244 | Gout |  |  | TT | GG |  |
| 15001245 | Gout |  |  | TT | GG |  |
| 15001246 | Gout |  |  | TT | GG |  |
| 15001247 | Gout |  |  | TT | AG |  |
| 15001248 | Gout |  |  | TT | GG |  |
| 15001249 | Gout |  |  | TT | AG |  |
| 15001250 | Gout |  |  | TT | GG |  |
| 15001251 | Gout |  |  | TT | GG |  |
| 15001252 | Gout |  |  | TT | AG |  |
| 15001253 | Gout |  |  | TT | GG |  |
| 15001254 | Gout |  |  | TT | AG |  |
| 15001255 | Gout |  |  | TT | GG |  |
| 15001256 | Gout |  |  | TT | GG |  |
| 15001257 | Gout |  |  | TT | GG |  |
| 15001258 | Gout |  |  | TT | AG |  |
| 15001259 | Gout |  |  | TT | AG |  |
| 15001260 | Gout |  |  | TT | GG |  |
| 15001261 | Gout |  |  | TT | GG |  |
| 15001262 | Gout |  |  | TT | GG |  |
| 15001263 | Gout |  |  | TT | AG |  |
| 15001264 | Gout |  |  | TT | AA |  |
| 15001265 | Gout |  |  | TT | AG |  |
| 15001266 | Gout |  |  | TT | AG |  |
| 15001267 | Gout |  |  | TT | GG |  |
| 15001268 | Gout |  |  | TT | GG |  |
| 15001269 | Gout |  |  | TT | GG |  |
| 15001270 | Gout |  |  | TT | AG |  |
| 15001271 | Gout |  |  | TT | GG |  |
| 15001272 | Gout |  |  | TT | AG |  |
| 15001273 | Gout |  |  | TT | GG |  |
| 15001274 | Gout |  |  | TT | AG |  |
| 15001275 | Gout |  |  | TT | AG |  |
| 15001276 | Gout |  |  | TT | AG |  |
| 15001277 | Gout |  |  | TT | AG |  |
| 15001278 | Gout |  |  | TT | GG |  |
| 15001279 | Gout |  |  | TT | GG |  |
| 15001280 | Gout |  |  | TT | AA |  |
| 15001281 | Gout |  |  | TT | GG |  |
| 15001282 | Gout |  |  | TT | GG |  |
| 15001283 | Gout |  |  | TT | GG |  |
| 15001284 | Gout |  |  | TT | GG |  |
| 15001285 | Gout |  |  | TT | AG |  |
| 15001286 | Gout |  |  | TT | AG |  |
| 15001287 | Gout |  |  | TT | GG |  |
| 15001288 | Gout |  |  | TT | GG |  |
| 15001289 | Gout |  |  | TT | AG |  |
| 15001290 | Gout |  |  | TT | GG |  |
| 15001291 | Gout |  |  | TT | GG |  |
| 15001292 | Gout |  |  | TT | GG |  |
| 15001293 | Gout |  |  | TT | GG |  |
| 15001294 | Gout |  |  | TT | GG |  |
| 15001295 | Gout |  |  | TT | AG |  |
| 15001296 | Gout |  |  | TT | GG |  |
| 15001297 | Gout |  |  | TT | AA |  |
| 15001298 | Gout |  |  | TT | GG |  |
| 15001299 | Gout |  |  | TT | AG |  |
| 15001300 | Gout |  |  | TT | AG |  |
| 15001301 | Gout |  |  | TT | GG |  |
| 15001302 | Gout |  |  | TT | AG |  |
| 15001303 | Gout |  |  | TT | GG |  |
| 15001304 | Gout |  |  | TT | GG |  |
| 15001305 | Gout |  |  | TT | GG |  |
| 15001306 | Gout |  |  | TT | AG |  |
| 15001307 | Gout |  |  | TT | GG |  |
| 15001308 | Gout |  |  | TT | GG |  |
| 15001309 | Gout |  |  | TT | GG |  |
| 15001310 | Gout |  |  | TT | AG |  |
| 15001311 | Gout |  |  | TT | GG |  |
| 15001312 | Gout |  |  | TT | GG |  |
| 15001313 | Gout |  |  | TT | GG |  |
| 15001314 | Gout |  |  | TT | GG |  |
| 15001315 | Gout |  |  | TT | AG |  |
| 15001316 | Gout |  |  | TT | AG |  |
| 15001317 | Gout |  |  | TT | GG |  |
| 15001318 | Gout |  |  | TT | GG |  |
